# Modeling the Depth Resolution of Translucent Layers in Confocal Microscopy

**DOI:** 10.1002/smsc.202400120

**Published:** 2024-06-25

**Authors:** Maximilian Maier, Thomas Böhm

**Affiliations:** ^1^ Forschungszentrum Jülich GmbH, Helmholtz‐Institute Erlangen‐Nürnberg for Renewable Energy (IEK‐11) 91058 Erlangen Germany; ^2^ Department of Chemical and Biological Engineering Friedrich‐Alexander‐Universität Erlangen‐Nürnberg 91058 Erlangen Germany

**Keywords:** computational modeling, confocal microscopy, depth resolution, point spread function, Raman

## Abstract

Confocal microscopy is an established technique with manifold applications that offers the capability to perform nondestructive through‐plane imaging. However, depth resolution typically decreases when focusing below the surface of a sample, which limits the applicability. A computational model is introduced that calculates the axial resolution, its decay, and the attenuation coefficient from confocal through‐plane scans of translucent layers. The model is benchmarked with different polymers and objectives (air, water, oil) using a confocal Raman microscope. The algorithm requires a single through‐plane scan that allows to identify the sample by signal intensity differences. It fits the point spread function of the objective at the top and bottom interface of the specimen to extract the resolution at both interfaces and the attenuation coefficient of the sample. It provides robust outputs on various and even multilayered samples if the signal‐to‐noise ratio of the input is sufficient and if the layers are planar and homogeneous. The algorithm of the model is provided open‐source for MATLAB and Python. Quantifying microscope resolution in through‐plane scans can improve image analysis in multiple fields, and this study is a comprehensive proof‐of‐concept for the presented model. It establishes an accessible tool to quantify the depth resolution of confocal microscopy.

## Graphical abstract

A model for quantifying the depth resolution and intensity decay of depth scans of confocal microscopy on translucent films is presented. A case study with Raman microscopy proves that the model provides physically reasonable results for a variety of samples and immersion media and even of multilayered samples, which aids in quantifying features in confocal depth scans.

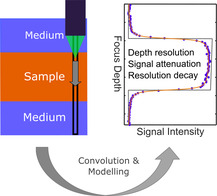

## 1 Introduction

Confocal laser scanning microscopy (CLSM) is a versatile microscopic technique. Its basic principle relies on spatially blocking out‐of‐focus light when collecting the signal. Thereby, the optical resolution and contrast can be increased substantially compared to wide‐field microscopic imaging.^[^ ^1^ ^]^ CLSM is implemented with a focused laser as an excitation source using a pinhole in the collecting beam path to block out‐of‐focus signal. CLSM can be subdivided based on the nature of the probed signal. The most common ones are confocal fluorescence microscopy (CFM) and confocal Raman microscopy (CRM). Both employ light in the UV–vis–NIR spectral range for exciting the sample. CRM collects the signal originating from Raman scattering (inelastic scattering of photons with optical phonons or molecular vibrations), whereas CFM probes fluorescent light stemming from the excitation of electronic transitions. Therefore, CLSM combines the high optical resolution of confocal microscopy with the high chemical sensitivity of Raman scattering or high contrast from fluorescence.

CLSM has manifold applications in various research fields, ranging from material science to life sciences.^[^ ^2^, ^3^, ^4^ ^]^ It has been applied to the study of 2D materials,^[^ ^5^ ^]^ the analysis of stress in oxides,^[^ ^6^ ^]^ and the microstructure of soft solid materials.^[^ ^7^ ^]^ In life sciences, CLSM has also found many use cases, such as imaging of living cells,^[^ ^8^ ^]^ precancerous lesions,^[^ ^9^ ^]^ or detection of single molecules in solution.^[^ ^10^ ^]^ CFM is predominantly used in life sciences by staining samples with fluorescent dyes,^[^ ^11^ ^]^ whereas CRM offers advantages in materials science.^[^ ^12^ ^]^ For instance, many polymers feature Raman active bonds. Thus, CRM has been used for analyzing polymers and their morphology. Examples include hydrogels^[^ ^13^ ^]^ or perfluorinated sulfonic acid membranes.^[^ ^14^ ^]^

CLSM offers various advantages over other microscopic techniques, like the improved contrast and resolution compared with wide‐field optical microscopy, and the capability to perform nondestructive through‐plane imaging on bulk samples, which is impossible for other approaches such as scanning electron microscopy. However, CLSM also faces drawbacks, such as limited lateral resolution compared with electron and scanning probe microscopy, and typically suffers from a loss in axial resolution with increasing focus depth in through‐plane scans. The latter is caused by changes in the refractive index at the interface to the immersion medium or when samples consisting of multiple materials are investigated. There are approaches available to mitigate the associated issues with poor contrast, low achievable imaging depth, and unreliable spatial dimensions of features in the images. For instance, adaptive optics can be applied to compensate for refraction‐induced optical artifacts,^[^ ^15^ ^]^ or imaging of sections instead of bulk samples can be employed to avoid these aberrations in the first place.^[^ ^16^ ^]^ However, these mitigation strategies require specialized equipment or information on the sample prior to imaging, which is not always available. To this end, an easy‐to‐use, software‐based solution that can provide an estimation or even a quantification of the severity of resolution and intensity losses in through‐plane scans can help microscopists in data analysis. Thus, we introduce a simple modeling tool to quantitatively investigate aberration‐induced resolution losses in confocal through‐plane imaging. We concentrate on the axial dimension in CLSM. A model for calculating the axial resolution is developed that carefully evaluates the experimental signal curve, which is a convolution of the point spread function (PSF) of the objective and the ideal composition profile of the sample and takes into account the loss of signal within the sample due to scattering and absorption.

The model was applied to CRM in a case study with different transparent polymer films. The algorithm yields high‐quality fits and extracts physically reasonable resolution parameters from confocal through‐plane scans for single‐layered samples. On top of that, we were able to quantify the resolution decay through multilayered composite polymer films with nonuniform refractive index profiles. We established a simple‐to‐use and open‐source accessible fitting procedure that fully characterizes the imaging system–sample combination of interest with a measurement as simple as a single confocal through‐plane scan.

### 1.1 Theoretical Framework

Determining the resolution of an imaging system is a nontrivial task. First, one must define the resolution of the optical configuration. The most common criterion used for lateral resolution is provided in Equation (1), which is based on the Rayleigh criterion for confocal microscopy. It states that two objects can be distinguished spatially when they are separated by half the diameter of the Airy disks formed at the object plane^[^ ^17^ ^]^

(1) $${\text{FWHM}}_{\text{lateral}}=\frac{0.4\lambda }{\text{NA}}$$

where NA is the numerical aperture of the objective and *λ* is the wavelength of the light. A more complex formula can be derived for the axial resolution if we consider an ideal confocal microscope with infinitely small pinhole^[^ ^1^ ^]^

(2) $${\text{FWHM}}_{\text{axial}}=\frac{0.64\lambda }{n-\sqrt{n^{2}-{\text{NA}}^{2}}}$$

where *n* is the refractive index of the immersion medium of the objective. However, just because two objects can be spatially resolved does not mean the different spectral features from the objects will not overlap as the scattering cross sections of different materials can vary. Therefore, resolution criteria used for imaging cannot be mapped one‐to‐one to microspectroscopy. This consideration is critical when the goal is to get a quantitative composition profile of the sample, where one must know how large the focal volume is and how much of the signal originates from within the focal volume, which is not only defined by the microscope but also by the investigated sample. Thus, it is evident that a single number cannot characterize the resolution of a confocal spectroscopic imaging system such as CRM.

If one is interested in correctly interpreting spectroscopic data from through‐plane scans, a precise understanding of the resolution variation in all three dimensions is mandatory. This work is concerned with optical depth profiling as mainly the axial resolution is impaired due to a varying refractive index profile when dealing with subsurface imaging and buried layers. Therefore, the problem simplifies to evaluating the change in axial resolution normal to the sample (*z*‐axis). Everall et al. have extensively characterized the phenomenon of worsening axial resolution in optical depth scanning.^[^ ^18^, ^19^, ^20^ ^]^ They explain the phenomenon with optical refraction at the interface between sample and immersion medium due to a mismatch of refractive indices in accordance with Snell's law. The ideal focal plane of the objective is not met any longer in this case. For light traveling through an interface where the refractive index increases, refraction at the interface induces a compression in axial direction and rays originating from different areas of the objective traverse the interface at different angles. Therefore, axial rays (from the central part of the objective) and marginal rays (from the outer part of the objective) are refracted to different points on the optical axis, thereby defocusing the laser and spreading the focal volume linearly in axial direction with increasing sample penetration depth. They define a depth of focus (dof): the distance between the focus points of the marginal and paraxial rays^[^ ^18^ ^]^

(3) $$\text{dof}=\left(\sqrt{\frac{{\text{NA}}^{2}\left(n^{2}-1\right)}{1-{\text{NA}}^{2}}+n^{2}}-n\right)z_{0}$$

where *z*_0_ is the nominal focus depth (i.e., vertical displacement of the microscope stage, the distance the objective's focal point has mechanically moved into the sample). *z*_0_ > 0 represents moving the laser focus below the surface. This model, solely based on refraction, overestimates the dof as it neglects the confocal aperture blocking part of the signal. Thus, the actual depth resolution of a confocal microscope is better than predicted by Equation (3) as the pinhole aperture decreases the effective focal volume. Baldwin et al.^[^ ^21^ ^]^ incorporated the aperture that blocks part of the signal into a geometrical model but overestimated the loss of intensity with depth. Sourisseau and Maraval^[^ ^22^ ^]^ integrated diffraction effects, such as off‐axis incident intensity distributions, diffraction integrals for calculating complex diffraction patterns for the transmission through the confocal aperture, or phase aberrations, and accurately predicted confocal through‐plane Raman responses for varying nominal focus depths, albeit with a computationally expensive model and without quantifying the decay in intensity and axial resolution through the sample.

A general model for image/signal formation in confocal microscopy describes imaging mathematically as the convolution of the PSF of the optical imaging system with the actual spatial composition profile of the sample. For spectroscopy performed with CLSM in through‐plane mode, this convolution equals to the experimentally collected signal along the optical axis. It is a weighted sum of photons originating from within the focal volume along the *z*‐axis. Mathematically, the signal can be expressed as^[^ ^1^ ^]^

(4) $$S(z)=\int_{-\infty }^{+\infty }s(y)L(z-y)dy=s(z)*L(z)$$

where *L*(*z*) is the instrumental PSF of the imaging system, *s*(*z*) is the ideal local intensity of the sample profile (i.e., the signal that would be acquired if the microscope had infinitely small resolution or, in other words, the actual spatial composition profile of the sample), and *y* is the variable of integration that represents the shift of the PSF for the calculation of the convolution.

The PSF describes the response of the confocal imaging system to a point source. It represents the degree of blurring in the image of a point object and, therefore, is a descriptor for the quality of the imaging system. It also translates into the minimal resolution a CLSM can achieve. As there are no real point sources, one can determine the PSF by acquiring the signal of a sample that is substantially smaller than the optical diffraction limit. When the axial PSF is of interest, a through‐plane scan across a very thin layer, ideally a 2D material, is well suited. A mathematical expression for the axial PSF is needed to model CLSM through‐plane data with Equation (4). In the literature, different functions have been applied to model the PSF of optical imaging systems, such as a Lorentzian^[^ ^23^, ^24^ ^]^ or a Gaussian profile.^[^ ^25^, ^26^ ^]^

In this work, we introduce a model that calculates the PSF of a confocal microscope based on experimentally acquired through‐plane scans through samples with sharp phase boundaries. This model extends the state‐of‐the‐art by including not only the PSF, but also the intensity decay (with the attenuation coefficient as descriptor) of the signal with increasing focus depth. The model provides a quantitative output on the axial resolution at different positions within a single confocal through‐plane scan.

## 2 Experimental Section

### 2.1 Sample Preparation

Three different polymer films were chosen to verify the theoretical and computational model: Nafion 211 (refractive index *n* = 1.35^[^ ^27^ ^]^) with a nominal thickness of 25.4 μm (Chemours, USA), a 23 μm thick polyethylene terephthalate (PET) layer (Chemours, USA; *n* = 1.58^[^ ^28^ ^]^), and a 16 μm thick polypropylene (PP) film (*n* = 1.49^[^ ^29^ ^]^). For measurements of thicker layers, a 78 μm thick PET film (Chemours, USA) and a 135 μm PP layer were used. The PET films were sent as protective layers for the Nafion 211 membranes and were detached prior to the measurements. The PP was in‐house fabricated at the institute of polymer materials at the University of Erlangen‐Nuremberg. These materials were chosen for their availability as polymer films with a specific thickness, their high transparency, and their suitability as Raman‐active materials. The thicknesses of the samples used for this study were verified with a micrometer gauge (IP 54, Mitutoyo, Japan). Unless stated otherwise, the PET, PP, and Nafion layers have the above‐specified thicknesses. Pieces of each material with a size of roughly 4 cm^2^ were cut from sheets for the measurements. Before each measurement, the samples were rinsed with 2‐propanol and deionized (DI) water (Nafion 211 only with DI water due to its excessive swelling in 2‐propanol).

For manufacturing composite multilayered samples, combinations of PET and Nafion 211 layers were carefully stacked on each other. The layers were merged by annealing the samples above the glass transition temperatures of Nafion (*T*_g_ = 130 °C)^[^ ^30^ ^]^ and PET (*T*_g_ = 70 °C)^[^ ^31^ ^]^ and applying constant pressure using a high‐performance press (LAB LINE P200S, COLLIN Lab & Pilot Solutions GmbH, Germany). The samples were hot‐pressed at 155 °C and 1.2 MPa for 6 min. Two different sequences for stacking the layers on each other were used: Nafion 211/PET/Nafion 211 and PET/Nafion 211/PET. These multilayered samples are labeled as Nafion–PET–Nafion and PET–Nafion–PET in Section 3.5.

A silicon wafer and monolayer graphene (trivial transfer graphene, ACS material, USA) deposited on a glass slide were used to determine the PSF of each objective. For the water and oil immersion objectives, a droplet of immersion oil or DI water was put on the sample.

### 2.2 CRM and Data Analysis

CRM was selected as a representative optical microscopic technique for this study. A Witec alpha300 RA (WITec GmbH, Germany) was employed for through‐plane confocal laser scanning. Three different objectives were employed for acquiring through‐plane scans: a W Plan‐Apochromat 63×/1.0 (magnification and NA) water immersion objective, an EC Epiplan‐Neofluar 100×/0.9 metallurgical objective, and a Plan‐Apochromat 63×/1.4 oil immersion objective (Carl Zeiss Jena GmbH, Germany). For water immersion measurements, a home‐built sample holder was used (Figure S1, Supporting Information), whereas for the oil immersion and ambient measurements, samples were measured on a glass substrate. The oil immersion objective required the use of a 170 μm thick cover glass between sample and oil film to account for the correction of the oil objective. The employed immersion oil was Immersol 518 F (Carl Zeiss Jena GmbH, Germany).

For excitation of the samples, a solid‐state laser emitting at 532 nm was used. The optical power of the laser was adjusted for every combination of immersion medium and type of polymer film. This was necessary to avoid beam damage, which impedes the Raman signal collection and often leads to an intolerable amount of fluorescent background. Nafion and PP were stable in all immersion media at up to the maximum power of the laser (50 mW). For PET, the laser power was adjusted to 30 mW for the oil immersion objective and the 100× metallurgical objective and to 40 mW for measurements in water immersion. The microscope operated in reflection mode. The collected Raman signal was analyzed with a WITec UHTS 300 VIS spectrometer equipped with a Peltier‐cooled back‐illuminated EMCCD camera (1600 pixels) and a 600 grooves mm^−1^ optical grating. The spectral resolution of this configuration is ≈3 cm^−1^. Axial scans of the samples were performed to obtain through‐plane profiles. At each point, a single Raman spectrum was acquired with an integration time of 200 ms. The integration time was a trade‐off between sufficient signal‐to‐noise ratio and fast scanning time. The through‐plane scans were extended over a depth around twice the thickness of the investigated sample layer. The axial step size was adjusted for each objective to keep the step size close to half the value of the full width at half maximum (FWHM) of objective's PSF, per the Nyquist–Shannon sampling theorem.^[^ ^32^ ^]^ Therefore, Δ*z* (step size of the axial through‐plane scan) was set to 500 nm for the 100× metallurgical objective and 666 nm for the water and oil immersion objectives. An axial step size of 200 nm was used for all objectives to determine the PSF. For each sample, four to five through‐plane scans were acquired at different positions on the sample.

The background signal of the raw Raman spectral data was subtracted with a shape‐based algorithm implemented in the Witec Project FIVE+ software. A shape size of 400 and a noise factor of 1 were used as parameters for the algorithm (shape size of 100 for the oil immersion data). The authors note that the presented computational model is not limited to data after postprocessing with WITec Project FIVE+. Any commercial or custom‐designed code capable of performing background subtraction of Raman spectra is equally suited.

### 2.3 Code Development

All further data treatment, the fitting procedure, and algorithms were implemented in custom‐built scripts. The code was implemented twice, with the open‐source programming language Python and the commercial package MATLAB (MathWorks, USA).

The code is divided into four files, a main script, and three functions executing the required specific computations. The script file performs the fitting procedure, calls all the necessary functions, and visualizes the optimized convolution for the given experimental data. First, a function or script is required for importing and processing the raw spectral data from the files exported by the microscope software. The spectral range for the sum filter is specified depending on the imaged material and immersion medium (Figure S2, Supporting Information), and the integrated intensity is min–max normalized. For a high‐quality fit of the through‐plane scan, a precise determination of the position of the interface(s) between different materials and immersion media within the scan data is of uttermost importance. The interface between two materials in the scan is observed as an intensity swap in the sum filter signals of the two materials. For an ideal, sharp transition between two materials, this intensity change occurs as a steep increase from zero to one or decrease from one to zero, and the transition between the two materials is mathematically defined as an inflection point where the second derivative of the profile equals zero. Therefore, we use a second‐derivative test with the experimental intensity data and a sigmoid function of the form

(5) $$f\left(z\right)=a+\frac{b}{1+e^{\pm c\left(z-d\right)}}$$

where *z* are the focal positions of the scan, and *a*, *b*, *c*, and *d* are free parameters of the sigmoid function. The value *d* represents the inflection point of the sigmoid function and, when fitted successfully, directly relates to the respective interface of the through‐plane intensity data. The sign of *c* (Equation (5)) depends on whether we fit an interface where the signal of interest increases or vanishes. After automatically fitting all interfaces of the experimental data, the function returns the through‐plane intensity data over focus depth, a set of positions for all interfaces, and the step size Δ*z*. If the second‐derivative test does not produce a high‐quality fit and therefore only yields unreliable interface positions (due to high noise, or extreme resolution and intensity decay), the program incorporates the option to manually set the interfaces from visual inspection of the through‐plane intensity data. Similarly, the program also offers the possibility to account for uneven intensity background below or above the sample (e.g., fluorescence, or spectral overlap between sample components and immersion medium). Thereby, the intensity value of the ideal profile above and beneath the interfaces is set to values calculated by averaging the first five and last five data points of the through‐plane scan, respectively.

The two remaining functions calculate the convolution function (see Section 3.2 and Equation (9) for details) and evaluate the residual sum of squares for the intensity data points and the calculated convolution. A parameter sweep is performed in the main script for all independent parameters of the convolution and the residual sum of squares is calculated for each iteration. The optimization procedure is implemented by minimizing the residual sum of squares for the parameter sweep. As a quality indicator for the optimum fit of the confocal through‐plane data, the coefficient of determination is calculated according to the following equation:

(6) $$R^{2}=1-\frac{\text{LSQ}}{{\text{SS}}_{\text{tot}}}$$

LSQ is the residual sum of squares determined from the fit, and SS_tot_ is the total sum of the squares of the experimental data points.

The computation time of the program depends on the through‐plane data input, the parameter sweep's number of iterations, and the spatial resolution of the convolution. Thicker layers (in consequence deeper through‐plane scans) require order of magnitude higher computation time than thin layers (when using 1500 iterations for the parameter sweep, a 25 μm thick Nafion layer requires 19 s and 135 μm PP a computation time of 492 s) as the iterative calculation of the convolution becomes more and more computationally expensive. A stepwise change of the parameter sweep's increment, first finding a rough estimate of the optimized parameters and then reducing the increment and decreasing the parameter sweep's range, reduces the computation time significantly in such cases. Parallelization of the iterative sweep was implemented to lower the computation time of the program. For all samples presented in this work, the computation time of the program (in MATLAB) was below 15 min on a standard desktop computer (AMD Ryzen 7 5800× 8‐core processor with 32 GB random access memory) if the parameter sweep was adjusted for the evaluated sample properties, e.g., thickness, multilayered or single‐layered.

### 2.4 Statistical Analysis

All confocal through‐plane scans were performed independently four times (three times for the composite samples in Section 3.5). The error bars represent the standard deviation calculated from the independent through‐plane scans (i.e., mean ± *σ*). Each figure shows one independent measurement and the corresponding fit. Repetitions for statistical analysis and to ensure reproducibility were put into the supplement. We refrain from performing statistical analysis (e.g., calculation of mean and standard deviation) on the attenuation factor for sample/immersion medium configurations where only negligible intensity decay (≤10^−3^ μm^−1^) over focus depth arises (Figure 2 and Table 2).

## 3 Results and Discussion

### 3.1 PSF of the Objectives Under Ideal Conditions

**Figure 1:**
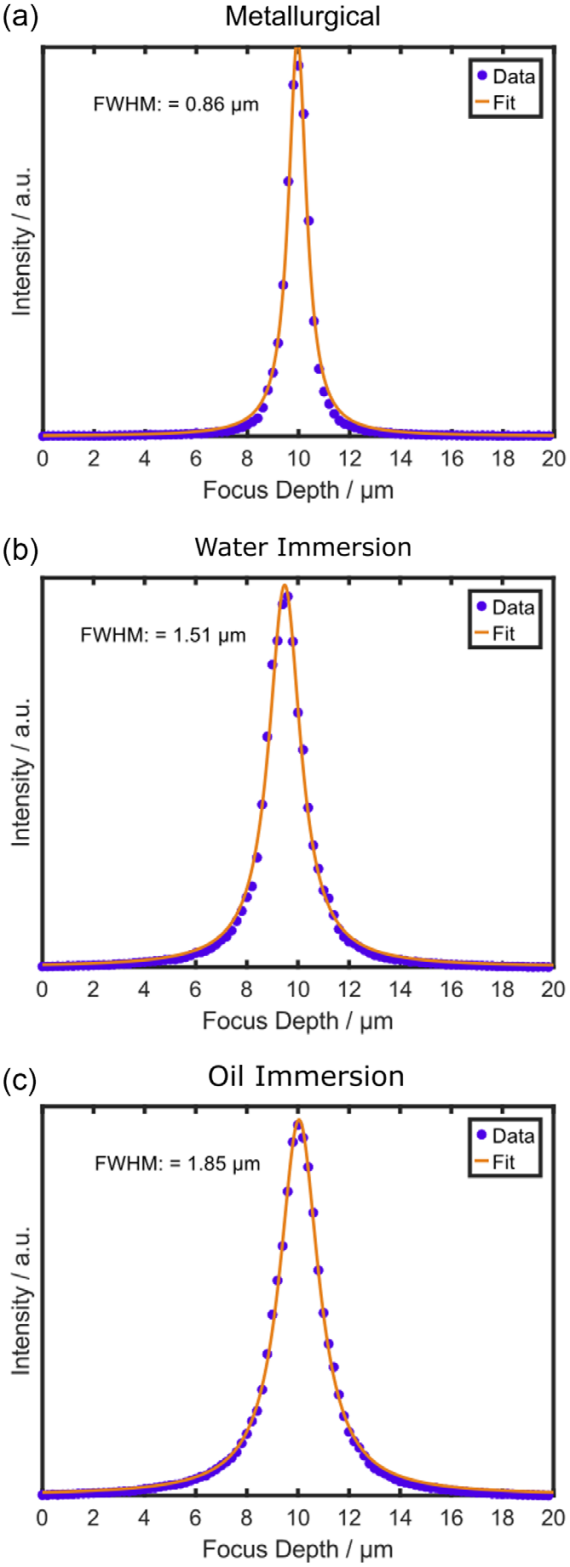
PSF of the a) 100×/0.9 metallurgical, b) 63×/1.0 water immersion, and c) 63×/1.4 oil immersion objective, evaluated by a confocal through‐plane scan of a silicon wafer. The axial step size was set to 200 nm. A sum filter in the region between 500 and 580 cm^−1^ was used to integrate the intensity of the silicon signal for each focal position. A Lorentzian (Equation (8)) was employed as a fitting function, and its FWHM represents the axial resolution of the objective.

First, the best possible diffraction‐limited axial resolution of each objective is determined to obtain a baseline for the objectives and the microscope setup under ideal conditions. It corresponds to the resolution one expects at the interface between immersion medium and the sample's surface, without artifacts caused by a sample when performing subsurface imaging. In this study, the diffraction‐limited axial resolution is defined as the FWHM of the integrated Raman signal of a confocal through‐plane scan of an optically thin layer. Optically thin equals a thickness or laser penetration depth that is substantially smaller than the diffraction‐limited resolution. We use a silicon wafer and monolayer graphene to quantify the axial PSF of the employed microscope objectives. In **Figure** **1**, the integrated intensity of the first‐order silicon band (sum filter from 500 to 580 cm^−1^) from exemplary scans of a silicon wafer is shown for the metallurgical, the water immersion, and the oil immersion objective. The data were fitted with a Lorentzian (Equation (8)). The FWHM represents the axial resolution of the objectives as the absorption depth of silicon at 532 nm wavelength is below 0.5 μm.^[^ ^33^ ^]^ The metallurgical objective features the highest axial resolution with 0.87–0.91 μm. The water immersion and oil immersion objectives exhibit lower resolutions with FWHMs of 1.46–1.6 μm and 1.83–1.85 μm (average values in **Table** **1**). Notably, the immersion objectives feature lower resolutions despite higher NA, which appears counter‐intuitive when considering that the theoretical resolution limit is governed by the NA of an objective (Equation (1) and (2)). However, the employed microscope contains a pinhole aperture of fixed size, which performs best with the metallurgical 100× objective. Objectives with lower magnifications would require smaller pinhole sizes to achieve the same resolution.^[^ ^34^ ^]^ Hence, the two 63× immersion objectives perform worse than expected from the simple equations. Further, manufacturing tolerances likely additionally affect the achievable resolution. The confocal through‐plane data for the graphene scans can be found in Figure S3, Supporting Information. The FWHM values of the PSFs from monolayer graphene and the silicon wafer are very similar (Table 1), which proves that both samples are well suited for deriving the axial resolution. This result is noteworthy as the optical thickness of a silicon wafer is substantially higher than for monolayer graphene.

**Table 1:** Axial resolution according to PSF determined for the 100×/0.9 metallurgical, the 63×/1.0 water immersion, and the 63×/1.4 oil immersion objective. The measurement was performed by through‐plane scanning of a silicon wafer or the monolayer graphene sample and by fitting the through‐plane scan with a Lorentzian (Equation (8)). At least five independent through‐plane scans (Figure 1 and S3, Supporting Information) were averaged. The error values represent the standard deviation calculated from the independent through‐plane scans.

Objective	FWHM_axial_ [μm] on silicon wafer	FWHM_axial_ [μm] on monolayer graphene
100×/0.9, metallurgical	0.87 ± 0.02	0.91 ± 0.02
63×/1.0, water immersion	1.46 ± 0.06	1.60 ± 0.10
63×/1.4, oil immersion	1.83 ± 0.08	1.85 ± 0.05

### 3.2 Model Development

Determining the axial resolution of the microscope on optically thin layers is a straightforward task, but obtaining this information from thicker samples requires a compensation for the artifacts that occur in confocal through‐plane scanning. Hence, a model has to be created that accounts for these artifacts in order to reliably quantify the resolution in realistic samples.

As described in Section 1.1, the measured through‐plane intensity is a convolution of the PSF of the optical system with the spatial composition profile of the sample. Therefore, the approach is to fit the experimental data with a convolution function and extract the physical parameters of interest (axial resolution, attenuation coefficient) from the respective components, which are the PSF and the composition profile. The convolution function contains four independent parameters: the FWHM values of the PSF at the upper and lower interface of the layer, a factor representing the intensity decay within the sample, and the offset of the maximum intensity of the ideal profile relative to the measured profile.

The exponential intensity decay with increasing focus depth within the sample is attributed to attenuation by scattering, absorption, and defocusing of the laser according to the Beer–Lambert extinction law

(7) $$I=I_{0}e^{-\mu z}$$

where *μ* is the exponential attenuation coefficient, and *I*_0_ is the maximum intensity of the confocal through‐plane scan. An offset from the observed experimental profiles to the theoretical maximum intensity is added to the model to account for the diffraction‐limited resolution of microscopy: At an infinitely small resolution, the maximum intensity of a material in a through‐plane occurs at the transition from the immersion medium to the specimen. However, the limited actual resolution results in a measured intensity peak that occurs up to several microns below the transition. The profile will show a maximum when the focal volume is filled with the material of interest when only negligible amounts of immersion medium account for the signal. Thus, the observed maximum intensity in experimental data is a convolution of the scattering cross section of the specimen with the PSF and the extinction law. A correct fitting of these functions to the measured data, therefore, requires an initial intensity offset to account for this effect. To summarize, the ideal composition profile is based on the determined (second derivative test) interfaces of sample and immersion medium (ideal sharp transitions), the factor representing the intensity decay within the sample, and the offset of the maximum intensity of the ideal profile relative to the measured profile.

The convolution is modeled with a linear resolution decay within the sample in accordance with optical refraction theory (Equation (3)). This resolution decay is implemented by assuming a linear increase of the FWHM of the PSF with sample penetration depth from the first to the second interface. The outputs of the model are the resolutions at the first and second interface and the attenuation coefficient within the scan. They are obtained by a least squares approach: The four parameters (FWHM at first and second interface, attenuation coefficient, and intensity offset) are swept, and the combination that results in the lowest value of the residual sum of squares between the fit and the measured data is provided as the output. These specific values for the FWHM of the PSF at the interfaces, the resolution decay per distance (the ratio of the difference of the FWHMs at the interfaces and the uncorrected optical thickness of the layer), and the attenuation coefficient μ are extracted as descriptors for the optical resolution (decay) and the translucence of the optical system–sample combination.

The PSFs of the CRM system used in this work are fitted best with a Lorentzian (Figure S4, Supporting Information). Hence, we use the following equation to express the PSF

(8) $$L\left(z\right)=\frac{1}{\pi }\frac{0.5\gamma }{z^{2}+{\left(0.5\gamma \right)}^{2}}$$

*γ* is the FWHM of the Lorentzian. *L*(*z*) is normalized so that $\int_{-\infty }^{+\infty }L\left(z\right)=1$. In this work, the axial resolution is defined as the FWHM of the PSF determined from our model.

The final formula in the model used to fit the intensity data is the convolution of the PSF with linearly decreasing resolution and the ideal spatial composition profile ($s\left(z\right)\;=\text{rect}\left(\frac{z}{t}\right)he^{-\mu z}$) with exponentially decaying intensity

(9) $$S(z)=s(z)*L(z)=\int_{-\infty }^{+\infty }\text{rect}\left(\frac{y}{t}\right)he^{-\mu y}L(z-y,\gamma (z))dy$$

where *L*(*z*) is the instrumental PSF of the imaging system exhibiting a FWHM *γ*(*z*) with a linear dependency on focus depth beneath the surface, $e^{-\mu z}$ describes the evolution of the ideal intensity profile through the sample (i.e., the signal that would be acquired if the microscope had infinitely small resolution or, in other words, the actual spatial composition profile of the sample), *h* is the offset of the theoretical ideal intensity at the first interface, and $\text{rect}\left(\frac{z}{t}\right)$ is a rectangular function which ensures that the ideal intensity is zero anywhere above or below the sample layer (*t* equals the measured optical thickness of the sample via the through‐plane scan, i.e., the difference in focus depth between the upper and lower interfaces of the sample and immersion medium).

### 3.3 Modeling Depth Resolution of Through‐Plane Scans Through Single‐Layered Samples

**Figure 2:**
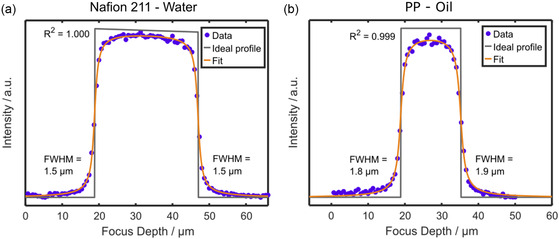
Modeling through‐plane scans with negligible refractive index mismatch between sample and immersion medium. a) Depth scan through Nafion 211 with the 63×/1.0 water immersion objective and b) through‐plane scan with PP and the 63×/1.4 oil immersion objective. The step size was set to 666 nm for both scans. Sum filters between 700 and 760 cm^−1^ for Nafion and from 2690 to 2770 cm^−1^ for PP were used for integrating the signal over depth. Fitting the raw data with the model automatically calculates the interface positions and the attenuation coefficient, and shows the convolution result as a fit superimposed on the raw data points. FWHM indicates the axial resolution at the upper and lower interfaces of the samples. The coefficient of determination was calculated according to Equation (6).

As a first step for validating the model, a physically simple configuration of a polymer film with a refractive index identical or close to that of the immersion medium was investigated. This setup allows to neglect spherical aberration as refraction at the interface between immersion medium and sample is minimal. Confocal through‐plane measurements of Nafion 211 with water as immersion medium (*n*_Nafion_ = 1.35 and *n*_water_ = 1.33) and PP with oil immersion (*n*_PP_ = 1.49 and *n*_Oil_ = 1.518) were investigated. **Figure** **2**a shows an exemplary fit of a Raman scan through Nafion 211 in water immersion, resulting in a depth resolution of 1.5 μm, which equals the expected value for this objective (Table 1). The FWHM of the PSF at the upper and lower interface is identical, meaning that there is no decay in axial resolution when performing through‐plane scanning with this specimen. Additionally, no significant intensity decay exists when scanning through the layer, which can be explained by the high transparency of this polymer and the insignificant refractive index difference.

The PP layer in oil immersion (Figure 2b) shows similar results, with the resolution at the upper and lower interface being nearly equal to the measured depth resolution of this objective. The result of an almost constant resolution in the depth scan is as expected for negligible refractive index mismatches. The evaluation was applied to three additional independent measurements of both samples (Figure S5, Supporting Information), showing that the fitting procedure models trough‐plane data of Nafion 211 in water and PP in oil with high quality (*R* ^2 ^≥ 0.996) and reliability (**Table** **2**). The additional scans of PP in oil (Figure S5b, Supporting Information) prove that the attenuation coefficient on average is the same as for Nafion in water (one scan of PP in oil with minimal attenuation is considered as an outlier), as expected for the minimal refractive index mismatch. Taken together, both configurations can be explained well with the model.

**Table 2:** FWHMs at the first and second interface as independent fit parameters determined for Nafion 211 and the 63×/1.0 water immersion objective, and PP with the 63×/1.4 oil immersion objective by fitting the through‐plane scan with the described model (see Section 3.2). The arithmetic mean and the standard deviation for four independent through‐plane scans are provided (Figure 2 and S5, Supporting Information).

Sample (thickness) and immersion medium	FWHM [μm] first interface	FWHM [μm] second interface	Attenuation coefficient *μ* [μm^−1^]
Nafion 211 (25.4 μm), water	1.53 ± 0.05	1.58 ± 0.10	–
PP (16 μm), oil	1.80 ± 0.09	1.90 ± 0.09	–

The thickness of the samples can be calculated from these scans when there is no mismatch in refractive indices. Here, the optical thickness must match the nominal thickness of the layer as samples in immersion media with negligible refractive index difference were analyzed. When subtracting the modeled focal positions of the first and second interfaces for the scans (Figure 2), a thickness of 29 μm is obtained for Nafion and a thickness of 16 μm for PP. The measurement of PP equals the nominal thickness of this sample. Nafion shows a higher value than its dry thickness of 25.4 μm, which is caused by the ≈10–15% swelling of Nafion in water.^[^ ^14^ ^]^

**Figure 3:**
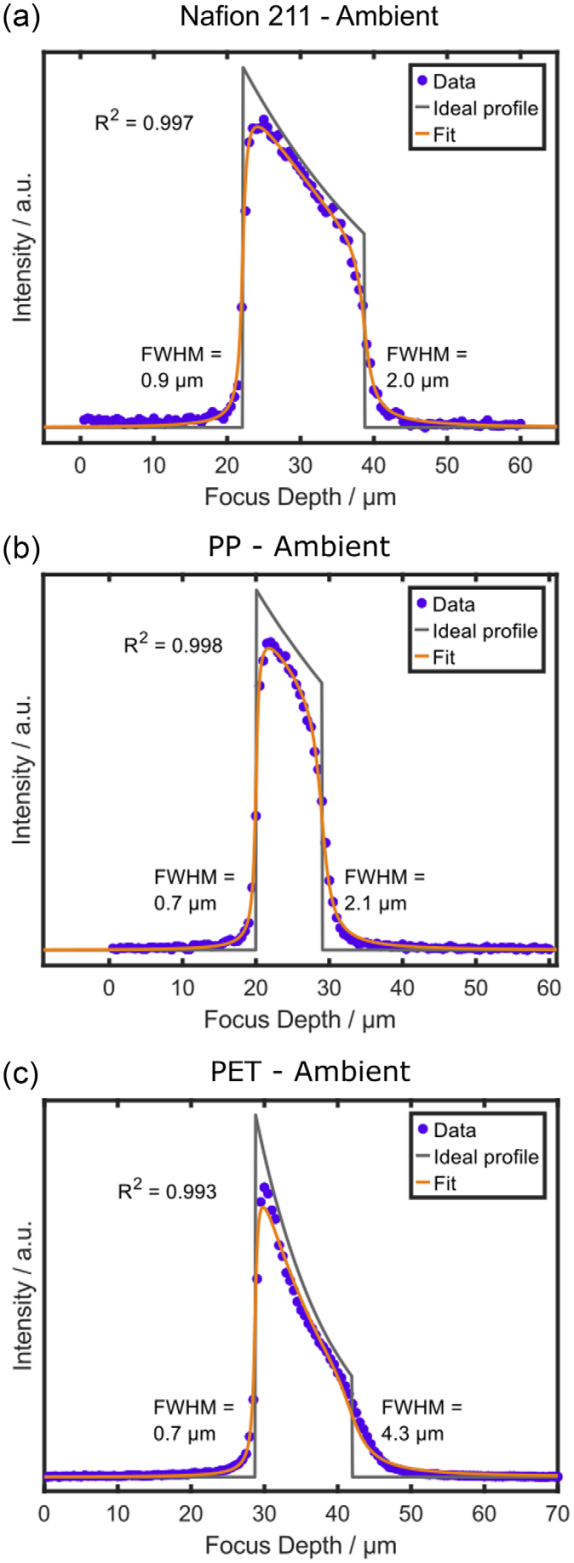
Modeling through‐plane scans for significant refractive index mismatches between sample and immersion medium. Depth scans were acquired with the 100×/0.9 metallurgical objective through a) Nafion 211, b) PP, and c) PET. The step size was set to 500 nm for all through‐plane scans. Sum filters of 700–760 cm^−1^ for Nafion, 790–840 cm^−1^ for PP, and 1570–1650 cm^−1^ for PET were used for integrating the signal over depth. Fitting the raw data with the model automatically calculates the interface positions and the attenuation coefficient, and shows the convolution result as a fit superimposed on the raw data points. FWHM indicates the axial resolution at the upper and lower interfaces of the samples. The coefficient of determination was calculated according to Equation (6).

Next, the fitting algorithm was used to model polymer layers with a significant refractive index mismatch at the interface to the immersion medium. Therefore, we analyzed through‐plane Raman scans of Nafion 211, PP, and PET with the metallurgical objective (**Figure** **3**) in ambient conditions, as all samples exhibit the largest index difference with air (*n*_air_ ≈ 1.0). All layers were fitted with high quality, i.e., the convolution follows the data points with coefficients of determination *R* ^2^ ≥ 0.99 in all measurements. Three independent measurements for each layer type can be found in Figure S6, Supporting Information, and all extracted parameters of the fits are provided in **Table** **3**. All samples feature significant intensity and resolution decay over focus depth. The resolution decay from the first to the second interface roughly scales with refractive index mismatch Δ*n* (higher Δ*n* exhibits larger resolution decay), with PET (*n* = 1.58) exhibiting an extreme resolution loss of 3.6 μm from the first to the second interface, PP (*n* = 1.49) a decrease of 1.4 μm, and Nafion 211 (*n* = 1.35) 1.1 μm. The layer thicknesses must be considered when correlating the resolution decay for the different films. Although Nafion 211 and PP show similar absolute resolution decays, the decay normalized to the optical thickness shows that PP has a higher resolution loss per focus depth (0.13 μm μm^−1^ vs 0.05 μm μm^−1^), as expected due to the higher refractive index of PP compared to Nafion. On the other hand, the significantly higher resolution decay for PET of 0.24 μm μm^−1^ cannot be solely explained by its only slightly higher refractive index compared to PP (1.58 vs 1.49). Therefore, the resolution decay extracted from our model for PET, besides classical refraction theory, must depend on additional light–matter interactions between the PET and the excitation laser or Raman‐scattered photons. For example, the partial crystallinity of PET^[^ ^31^ ^]^ might create additional defocusing of the laser or an increased scattering cross section for the Raman signal along a preferential plane.^[^ ^35^, ^36^, ^37^ ^]^ Notably, the model is able to fit the experimental data of PET well, although the exact origin of the pronounced resolution decay remains to be elucidated.

**Table 3:** FWHMs at the first and second interface, the resolution decay per distance, and the attenuation coefficient as independent fit parameters determined by fitting the through‐plane scans acquired with the 100×/0.9 metallurgical objective on Nafion 211, PP, and PET with the described model (see Section 3.2). The arithmetic mean and the standard deviation for four independent through‐plane scans are provided (Figure 3 and S6, Supporting Information).

Sample (thickness) and immersion medium	FWHM [μm] first interface	FWHM [μm] second interface	Resolution decay per distance [μm μm^−1^]	Attenuation coefficient *μ* [μm^−1^]
Nafion (25.4 μm), ambient air	0.88 ± 0.10	1.83 ± 0.15	0.05 ± 0.01	(3.62 ± 0.10) × 10^−2^
PP (16 μm), ambient air	0.70 ± 0.00	1.83 ± 0.21	0.13 ± 0.03	(3.91 ± 0.74) × 10^−2^
PET (23 μm), ambient air	0.70 ± 0.00	3.90 ± 0.28	0.24 ± 0.03	(9.45 ± 0.35) × 10^−2^

The extracted FWHM at the first interface for PP (Figure 3b) and PET (Figure 3c) is 0.7 μm, which is slightly below the axial FWHM of the metallurgical objective (≈0.9 μm). Additional measurements on these samples verified this result (Figure S6b,c, Supporting Information), which renders an inaccuracy of the fit unlikely. On the other hand, the FWHM of through‐plane scans of Nafion (Figure 3a and S6a, Supporting Information) reveal an FWHM at the first interface between 0.8 to 1.0 μm, which is in excellent agreement with the axial FWHM of the objective. Thus, there is no general offset for the model, but the differences in the scans of PP and PET are sample‐specific. A possible explanation for the deviation on these two samples is the high refractive index mismatch at the interface, which compresses the measured optical thickness according to the simple paraxial approximation^[^ ^38^ ^]^

(10) $$z_{\text{focus}}=\Delta n\times z_{0}$$

where *z*_0_ is the apparent focus point based on the mechanical displacement of the objective in axial direction (identical to the definition in Equation (3)), *z*_focus_ is the actual mean focal depth of the laser focus, and Δ*n* is the refractive index ratio between sample and immersion medium. For samples with large deviations from the immersion medium, the focus point is moving significantly deeper into the sample than expected from the mechanical displacement (in the case of PP and PET around 1.5 times), leading to an apparent resolution at the first interface which is slightly higher than the best possible resolution expected from the objective's axial PSF.

**Figure 4:**
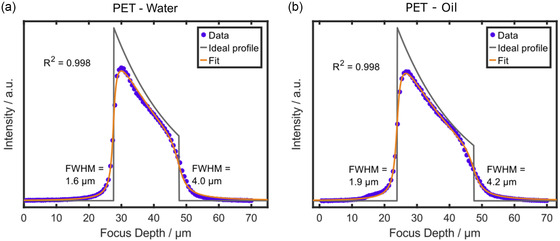
Modeling through‐plane scans through PET with immersion objectives. Depth scan through PET with the a) 63×/1.0 water immersion objective and b) 63×/1.4 oil immersion objective. The step size was set to 666 nm for both cases. Sum filters of 1570–1650 cm^−1^ for water immersion and 1602–1672 cm^−1^ for oil immersion were used for integrating the PET signal over depth. Fitting the raw data with the model automatically calculates the interface positions and the attenuation coefficient, and shows the convolution result as a fit superimposed on the raw data points. FWHM indicates the axial resolution at the upper and lower interfaces of the samples. The coefficient of determination was calculated according to Equation (6). The second interface of the measurement (b) in oil immersion has been set manually.

**Figure** **4** shows a confocal through‐plane scan of the PET film with the water immersion and the oil immersion objective. The FWHM at the first interface for PET (Figure 4) agrees with the axial FWHM of both objectives with an error margin of 0.1 μm (Table 1). Both configurations exhibit immense losses of intensity and resolution over thickness (**Table** **4**). Spherical aberration caused by the refractive index mismatch in this configuration at least partially explains this phenomenon for the water immersion objective, but it is unexpected for oil immersion due to the substantially smaller refractive index mismatch (1.58 and 1.518). The resolution decay for water immersion (0.13 ± 0.02 μm μm^−1^) is only slightly higher than for oil immersion (0.11 ± 0.01 μm μm^−1^) and the attenuation coefficient is almost identical as well (Table 4). Thus, if the loss of signal and resolution was only based on optical refraction at the interfaces, PET with oil immersion would exhibit drastically less decay than with water immersion. Therefore, the stronger decrease in signal strength and resolution for the oil immersion objective scan must have a different origin.

**Table 4:** FWHMs at the first and second interface, the resolution decay per distance, and the attenuation coefficient as independent fit parameters determined for a PET film measured with the 63×/1.0 water immersion objective and the 63×/1.4 oil immersion objective by fitting the through‐plane scan with the described model (see Section 3.2). The arithmetic mean and the standard deviation for four independent through‐plane scans are provided (Figure 4 and S7, Supporting Information).

Sample (thickness) and immersion medium	FWHM [μm] first interface	FWHM [μm] second interface	Resolution decay per distance [μm μm^−1^]	Attenuation coefficient *μ* [μm^−1^]
PET (23 μm), water	1.58 ± 0.05	4.13 ± 0.23	0.13 ± 0.02	(4.88 ± 0.15) × 10^−2^
PET (23 μm), oil	1.83 ± 0.10	4.25 ± 0.21	0.11 ± 0.01	(4.78 ± 0.09) × 10^−2^

Similarly like for the case of PET in air (Figure 3c), unaccounted interactions between the PET and the excitation of Raman scattering may provoke an intrinsic loss of signal and resolution irrespective of the immersion medium. This hypothesis is backed up by the fact that the PET layer features an FWHM of around 4 μm at the second interface, irrespective of the immersion medium. An explanation for the deviating result of the oil immersion configuration is a distortion of the through‐plane scan data by the spectral overlap of Raman bands of the immersion oil with the integrated peak of PET (Figure 4b and S7b, Supporting Information). Above the first interface there is a plateau with higher‐than‐normal background signal before transitioning into the sharply increasing signal at the interface. This plateau, albeit less pronounced, can also be found below the second interface. A completely separated spectral Raman band for PET and the immersion oil does not exist, and a thin layer of immersion oil between cover glass and sample is required for the oil immersion objective to prevent air inclusions. Therefore, the integrated signal does not purely stem from PET but also contains some Raman signal from the oil, leading to the nonuniform through‐plane scan. The model does not take such artifacts into account. Therefore, the fit for PET in oil, even though resulting in a high quality, might lead to wrong conclusions for the resolution and intensity decay. This phenomenon emphasizes the importance of carefully adjusting the sum filter for the intensity data of the specific immersion media/sample configuration of interest.

Despite these inconsistencies for the PET sample, the paraxial approximation (Equation (10)) for the measured sample thickness yields correct projections. The noncorrected optical thickness of the PET sample from the scans in water equals ≈19–20 μm (Figure 4a and S7a, Supporting Information) and ≈23–24 μm from the scans in oil (Figure 4b and S7b, Supporting Information), which is close to the nominal thickness of the PET layer (23 μm). Compensating the optically measured thickness for PET in water with the refractive index mismatch yields nearly identical values to oil immersion with ≈22.5–24 μm. Correcting the measurements with the metallurgical objective (optical thickness ≈13.5–14.0 μm, see Figure 3c and S6c, Supporting Information) yields ≈21–22 μm, which is in good agreement with the nominal thickness as well. It is striking that the simplest approximation of optical ray tracing to account for refraction effects yields good results, even though the PET layer showed counterintuitive results regarding the loss of intensity and resolution.

### 3.4 Limits of the Computational Model

**Figure 5:**
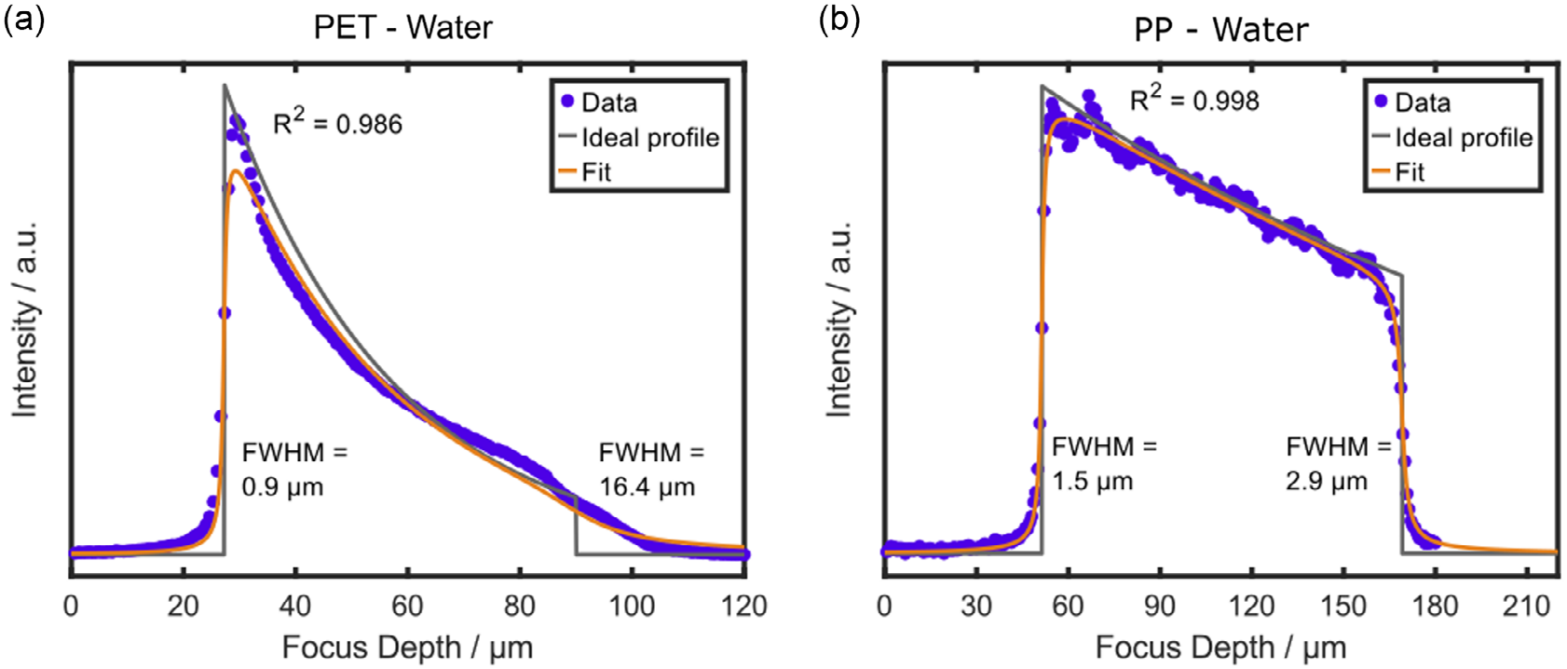
Modeling through‐plane scans for polymer films with larger thickness. Depth scans were acquired with the 63×/1.0 water immersion objective through a) PET (78 μm) and b) PP (135 μm). The step size was set to 666 nm for all through‐plane scans. Sum filters of 790–840 cm^−1^ for PP and 1570–1650 cm^−1^ for PET were used for integrating the signal over depth. Fitting the raw data with the model automatically calculates the interface positions and the attenuation coefficient, and shows the convolution result as a fit superimposed on the raw data points. FWHM indicates the axial resolution at the upper and lower interfaces of the samples. The coefficient of determination was calculated according to Equation (6). The second interface of the measurement of (a) PET has been set manually.

The resolution model provides accurate results for thin (<30 μm) samples. However, the signal quality fades with increasing focus depth, and to evaluate the limits of the model, thicker samples were investigated. Scans through PET showed significant loss of signal and resolution for all objectives even for a thin layer of just 23 μm. Therefore, a roughly threefold thicker PET layer (≈78 μm) was measured with the water immersion objective (**Figure** **5**a). Here, the fit of through‐plane intensity data shows poor quality with *R* ^2^ < 0.99. The model fails at correctly fitting the first interface, resulting in an FWHM at the first interface that is significantly smaller than the axial FWHM of the water immersion objective as the convolution does not match the datapoints. Equally, the intensity decay is not well represented by the convolution, which also results in a poor representation of the second interface.

Additional measurements on the thick PP and PET layers (**Table** **5** and Figure S8a, Supporting Information) show that the algorithm finds nonreproducible depth resolutions for the second interface this sample‐objective combination. Notably, the model is not generally limited to thin samples. Control measurements (Figure 5b and S8b, Supporting Information) on an even thicker PP layer (≈135 μm) prove that the model can fit thicker films with high accuracy. The PP layer shows significant less decay of signal intensity and resolution compared with the PET layer, despite its higher thickness. Thus, the model's boundary is not given by the thickness of the PET layer but by the poor data quality from highly scattering or absorbing samples, or from a nonconstant background. Further, the model is only valid for phase transitions that occur perpendicular to the optical axis of the microscope. Complex nonhomogeneous layers, e.g., fiber mats, or polymer blends with random 3D structure, cannot be predicted. Modeling the depth resolution for such layers requires more complex calculations than optical ray theory at planar interfaces and is beyond the scope of this work.

**Table 5:** FWHMs at the first and second interface, the resolution decay per distance, and the attenuation coefficient as independent fit parameters determined by fitting the through‐plane scans acquired with the 63×/1.0 water immersion objective on PET (78 μm) and PP (135 μm) with the described model (see Section 3.2). The arithmetic mean and the standard deviation for four independent through‐plane scans are provided (Figure 5 and S8, Supporting Information).

Sample (thickness) and immersion medium	FWHM [μm] first interface	FWHM [μm] second interface	Resolution decay per distance [μm μm^−1^]	Attenuation coefficient *μ* [μm^−1^]
PET (78 μm), water	0.90 ± 0.00	16.4 ± 3.60	0.24 ± 0.06	(3.38 ± 0.07) × 10^−2^
PP (135 μm), water	1.58 ± 0.23	2.53 ± 0.30	0.01 ± 0.01	(5.80 ± 0.96) × 10^−3^

Additionally, the user must find a reasonable trade‐off between scan acquisition time and sufficient signal‐to‐noise ratio. For weak Raman scatterers, the inherently weak signal can be compensated by an extended integration time. However, this leads to longer scanning time, which increases the risk of sample drift during the scan, leading to a systematic deviation in the acquired data. An adjustment of the increment step size of the scan, the integration time, and the *z*‐range of the through‐plane scan is mandatory to ensure sufficient signal‐to‐noise ratio with as short as possible acquisition time. These issues are less pronounced in CFM because significantly shorter integration times are possible due to the orders of magnitude stronger fluorescence response in comparison with Raman scattering.

### 3.5 Modeling Depth Resolution of Through‐Plane Scans Through Composite Polymer Films

**Figure 6:**
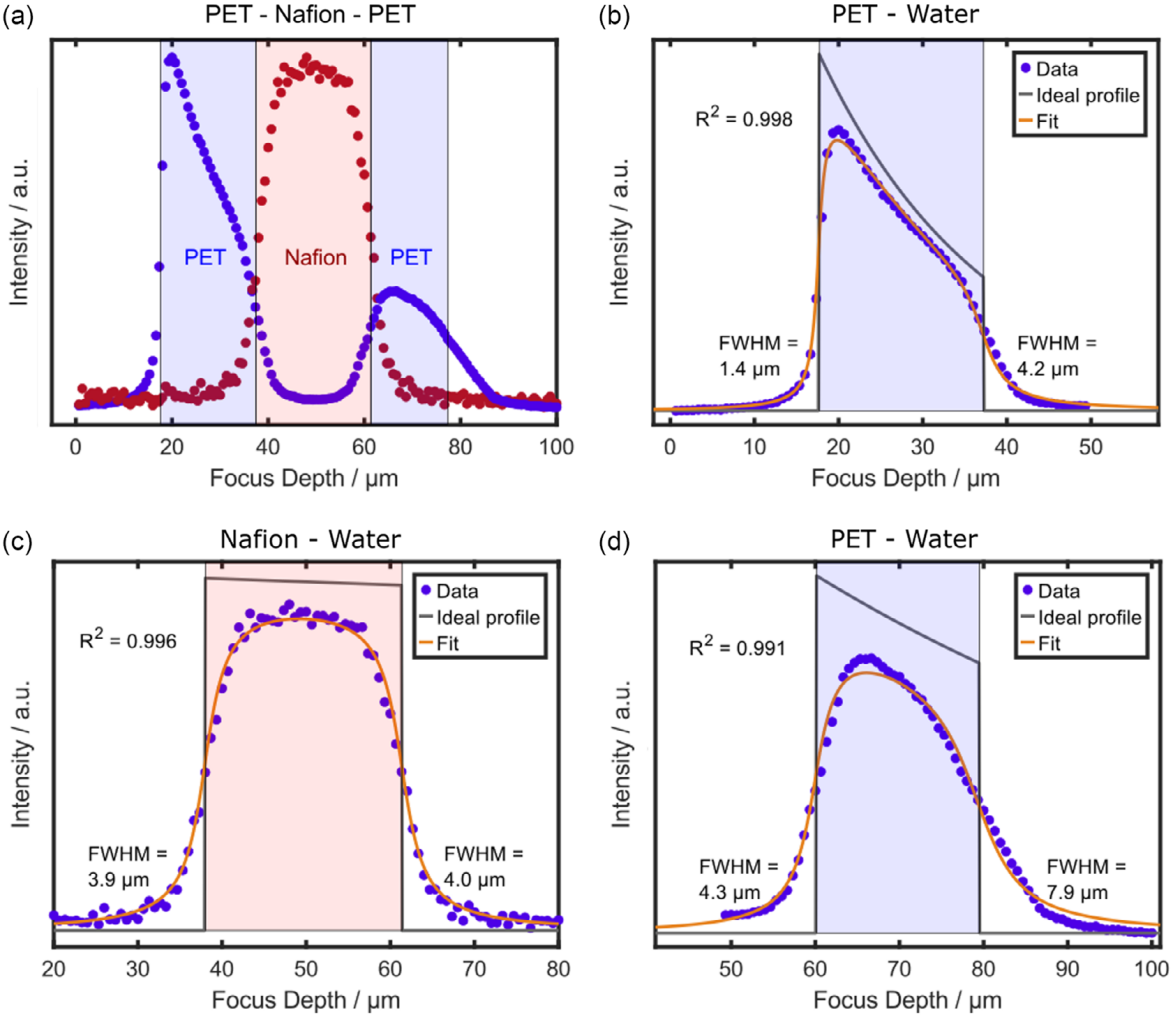
Modeling a through‐plane scan for a multilayered sample consisting of PET with a Nafion 211 interlayer. a) Integrated intensity of the Nafion and PET component signal over focus depth acquired with the 63×/1.0 water immersion objective using a sum filter of 720–740 cm^−1^ for Nafion (represented in red) and 1570–1650 cm^−1^ for PET (represented in blue). Modeling the through‐plane scan of b) the upper PET layer, c) the Nafion 211 interlayer, and the d) PET bottom layer. Shaded background colors indicate the interfaces between adjacent layers and their respective fits and are just meant as a guide‐to‐the‐eye. The step size was set to 666 nm for all through‐plane scans. Fitting the raw data with the model automatically calculates the interface positions and the attenuation coefficient, and shows the convolution result as a fit superimposed on the raw data points. FWHM indicates the axial resolution at the upper and lower interfaces of the samples. The coefficient of determination was calculated according to Equation (6). The second interface of the d) bottom layer of PET has been set manually.

After establishing the computational model for single‐layered samples, the follow‐up step is a proof‐of‐concept for accurately fitting the evolution of the depth resolution in multilayered composite samples. Stacking different materials on top of each other leads to more interfaces with refractive index differences at each transition and represents a more complex case for the model. A sandwich of PET–Nafion–PET was analyzed to evaluate this case. **Figure** **6**a shows the sum‐filter‐based through‐plane line scan of the two different components of the hot‐pressed polymer film stack, consisting of two 23 μm thick PET layers with a Nafion 211 (25.4 μm) interlayer. The measurements on composite films were performed with the water immersion objective due to its high signal‐to‐noise ratio. Nafion and PET exhibit spectrally separated peaks. Therefore, two sum filters can be used straightforward to differentiate the different layers.

The composite sample was analyzed by the resolution model by fitting the three layers independently. Figure 6b–d shows the fit of each adjacent layer and the resulting parameters are provided in **Table** **6**. As expected, the upper PET layer (Figure 6b) yields almost identical parameters compared to single‐layered PET in water (Figure 4a). The Nafion 211 interlayer (Figure 6c) results in an FWHM of 3.9 μm at the first interface, which is reasonably close to the FWHM of the adjacent second interface of the upper PET layer, given the resolution step size of the parameter sweep of the model (0.1 μm). The Nafion layer provokes no significant intensity or resolution decay, as it is expected from Nafion in water. The depth resolution at the bottom layer of PET (Figure 6d) follows the trend from the upper layer, starting with an FWHM of 4.3 μm that decreases to 7.9 μm at the last interface. Notably, the intensity of the PET signal does not fully reach the baseline within the Nafion interlayer, which is caused by the limited resolution at this penetration depth in the sample (more than 2.5× worse resolution than the ideal PSF of this objective). Consequently, the scan of the lower PET layer shows an increased intensity level in front of the upper interface. This effect is less pronounced for the upper layer of PET and is due to the necessary renormalization of the intensity data of the second layer to account for intensity losses between the first and the second PET layer. Thus, uneven intensity levels above or beneath the second layer are elevated.

**Table 6:** FWHMs at the first and second interface, the resolution decay per distance, and the attenuation coefficient as independent fit parameters determined by fitting the through‐plane scans of the multilayered PET–Nafion‐PET sample acquired with the 63×/1.0 water immersion objective and fitting each respective layer with the described model individually (see Section 3.2). The arithmetic mean and the standard deviation for three independent through‐plane scans are provided (Figure 6 and S9, Supporting Information).

Layer (thickness) and immersion medium	FWHM [μm] first interface	FWHM [μm] second interface	Resolution decay per distance [μm μm^−1^]	Attenuation coefficient *μ* [μm^−1^]
PET (23 μm), water	1.33 ± 0.12	4.00 ± 0.27	0.14 ± 0.02	(5.19 ± 0.45) × 10^−2^
Nafion (25.4 μm), water	4.17 ± 0.26	4.20 ± 0.20	0.00 ± 0.02	(1.48 ± 1.10) × 10^−3^
PET (23 μm), water	4.33 ± 0.06	8.13 ± 0.98	0.20 ± 0.05	(1.51 ± 0.28) × 10^−2^

The loss in resolution per distance is not identical between the first and second PET layer (Table 6), despite assumption of a linear progression of resolution decay with increasing focus depth. A possible explanation for this offset is the multiple interfaces between media of different refractive indices in this composite sample. Any interface that is nonperpendicular to the optical axis adds refraction artifacts that can affect the resolution additionally to spherical aberration, and in multilayered samples, the risk for this effect is higher than in single‐layer samples. Hence, the resolution decay for the first PET layer is equal to the single‐layer PET sample, but the bottom PET layer shows an increased resolution decay per distance (Table 6). In general, when looking at the complete scan, each layer of the composite sample is fitted with high accuracy (*R* ^2^ > 0.99) (Figure 6 and S9, Supporting Information). The depth resolution is tracked for the whole through‐plane scan and fitting the respective interfaces of each layer pairwise proves to be a good simplification. The opposite stacking order of a Nafion 211 and PET composite was analyzed as well (Figure S10, Supporting Information). The confocal through‐plane data were fitted successfully, extracting physically reasonable resolution parameters and attenuation coefficients, further proving the prowess of the presented model to extract the resolution in multilayered samples with varying refractive index profile. Notably, the resolution decay of the central PET layer (Table S1, Supporting Information) in this configuration is in the order of single‐layer PET samples (Table 4 and 6) as the refractive index mismatch between the immersion medium (water) and the upper Nafion layer is negligible. Thus, in this configuration, there is no interface above the PET layer that could add noticeable artifacts from refraction.

## 4 Conclusion

In this work, a computational model for evaluating the depth resolution of translucent samples in confocal microscopy is introduced. The model deconvolutes the PSF of the imaging system from the ideal sample profile by accounting for the main contributions of limited through‐plane imaging performance in confocal microscopy: refractive index mismatches and intensity losses in subsurface imaging. Within a few minutes of computation time, the algorithm provides reliable data on axial resolution and resolution decay with increasing focus depth, and can even be used to calculate layer thicknesses when the refractive index of the sample material is known. Future work might build on this algorithm by moving from a simple parameter sweep to a more advanced optimization method (possibly supported by machine learning), thereby reducing the number of iterations necessary and significantly reducing the computation time, especially for thick samples with a high number of layers.

The model can be applied to single‐layer samples as well as composite materials consisting of multiple layers. As long as the composite features spectrally distinct components, each layer can be fitted individually, yielding a precise quantification of the evolution of depth resolution through the sample. Limiting cases are given when high light attenuation and/or thick samples are investigated that result in poor signal‐to‐noise ratio and extreme resolution and intensity decay. Also, the model is restricted to homogeneous planar layers and not suited for, e.g., fibrous meshes embedded in a polymer. The extracted parameters crucially depend on the automatically determined focal positions of the interfaces, which are prone to miscalculation when the data contains noise, or the resolution decay is too extreme.

This study is a thorough proof‐of‐concept for the developed fitting algorithm. Especially the capability to analyze composite samples consisting of stacked films is advantageous for subsurface optical spectroscopy or imaging because this sample type is commonly used, e.g., for electrochemical energy systems,^[^ ^39^, ^40^ ^]^ wastewater treatment,^[^ ^41^ ^]^ gas separation,^[^ ^42^ ^]^ packaging,^[^ ^43^, ^44^ ^]^ and many more. The presented model provides a straightforward analytical tool for quantifying the axial resolution of a microscope through the whole sample depth. Obtaining reliable values of depth resolution of confocal through‐plane scans avoids misinterpretation of data, for instance, when small features in an image need to be characterized, or when analyzing spectra and weighting the spectral contributions of different components. This work shows that this model can be readily applied to CRM, but the underlying theory also holds true for other microscopic approaches, such as CFM.

To summarize, we provide an accessible tool that can be applied to different microscopic setups and adjusted to the required use case. Quantifying the depth resolution in a confocal through‐plane scan can substantially improve the analysis and interpretation of the data, potentially impacting the research on advanced composite material systems.

## Conflict of Interest

The authors declare no conflict of interest.

## Supporting information

Supplementary Material

## Data Availability

The data that support the findings of this study are available from the corresponding author upon request. The code developed in the course of this study is attached in the supporting information (both as MATLAB or Python code).
