# Supplementary material for: Modeling the Depth Resolution of Translucent Layers in Confocal Microscopy
Source: Small Sci. 2024 Jun 25;4(9):2400120. doi: 10.1002/smsc.202400120 (PMC11935062; doi:10.1002/smsc.202400120)
Supplement: Supplementary file 1 — Supplementary Material [file SMSC-4-2400120-s001.zip › smsc202400120-sup-0001-SuppData-S1.pdf]

Supplementary Material to

Modelling the Depth Resolution of Translucent Layers in Confocal Microscopy

Maximilian Maier[1,2], and Thomas Böhm[1,*]

1 Forschungszentrum Jülich GmbH, Helmholtz-Institute Erlangen-Nürnberg for Renewable Energy (IEK-11), Erlangen, Germany

2 Department of Chemical and Biological Engineering, Friedrich-Alexander-Universität Erlangen-Nürnberg, Erlangen, Germany

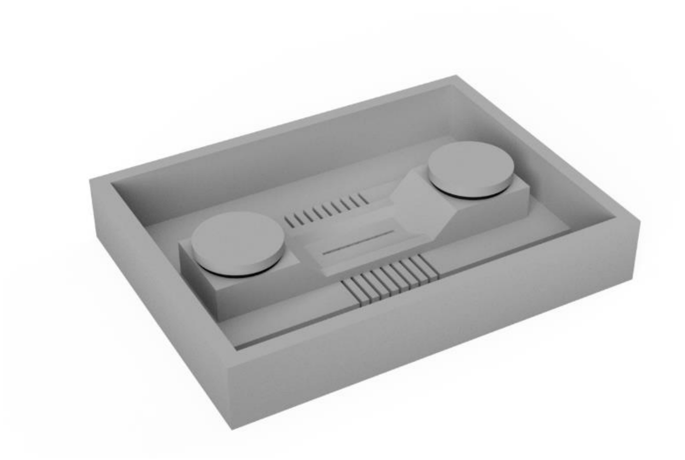

Figure S1: CAD model of the sample holder for the water immersion measurements. A magnetic clip is used to fix the membrane. The membrane is in contact with a water reservoir below and above the membrane due to the carved slots at the bottom.

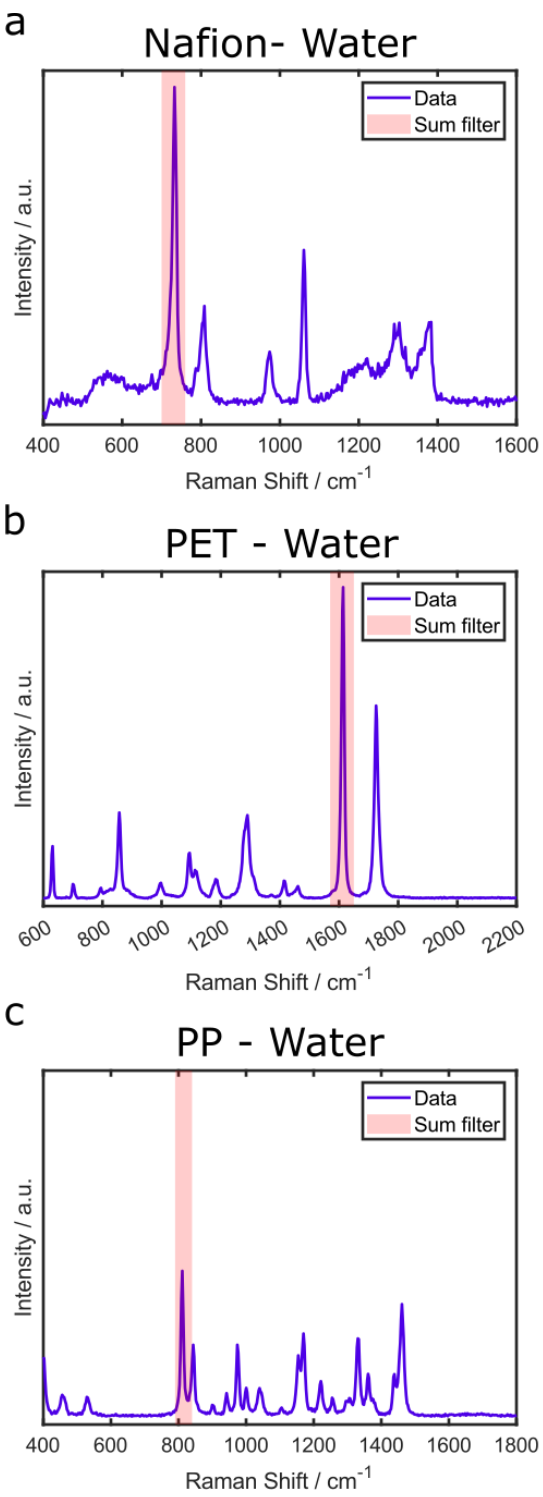

Figure S2: Raman spectrum of a) Nafion, b) PET, and c) PP acquired with the water immersion objective. The shaded area emphasizes the sum filter range of the respective polymer in water. The sum filter is employed to integrate the intensity of the signal for each focal position.

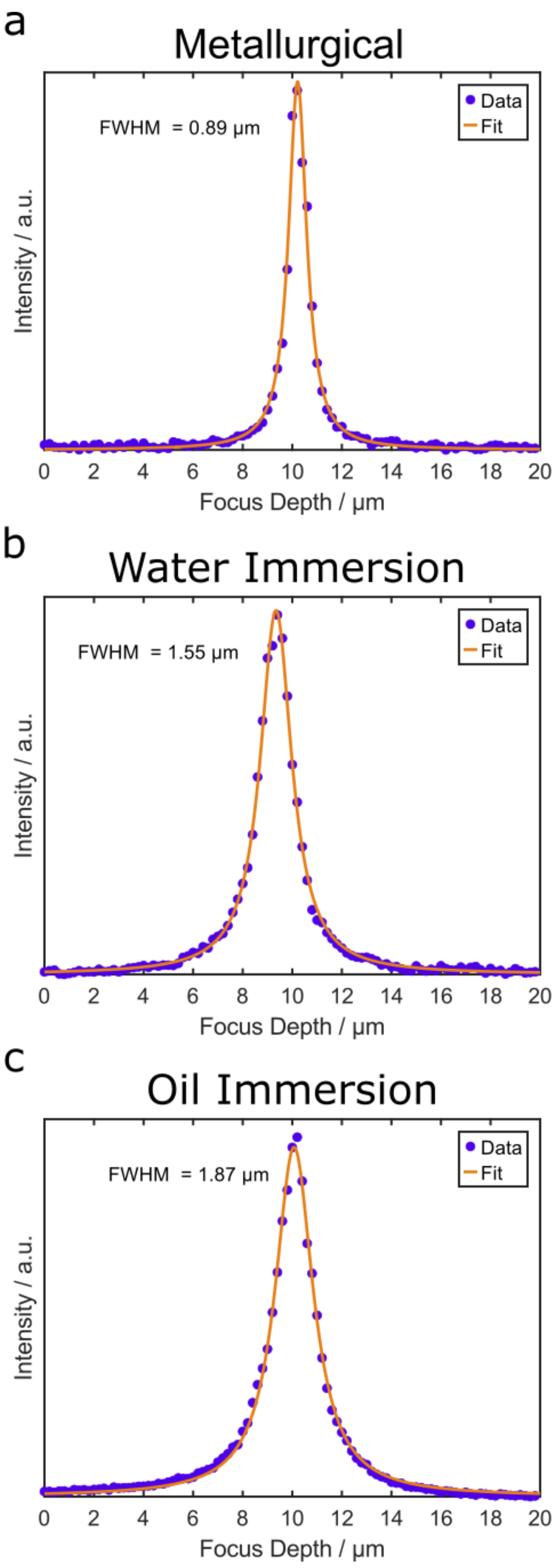

Figure S3: PSF of the a) 100x/0.9 metallurgical, the b) 63x/1.0 water immersion objective, and the c) 63x/1.4 oil immersion objective, evaluated by a confocal through-plane scan of a single-layer graphene sample. The axial step size was set to 200 nm. A sum filter in the region between 2600 cm^{-1} and 2800 cm^{-1} (2630 to 2710 cm^{-1} for oil immersion) was used to integrate the intensity of the 2D-peak signal for each focal position. A Lorentzian (**Equation 8)** was employed as a fitting function, and its FWHM represents the axial resolution of the objective.

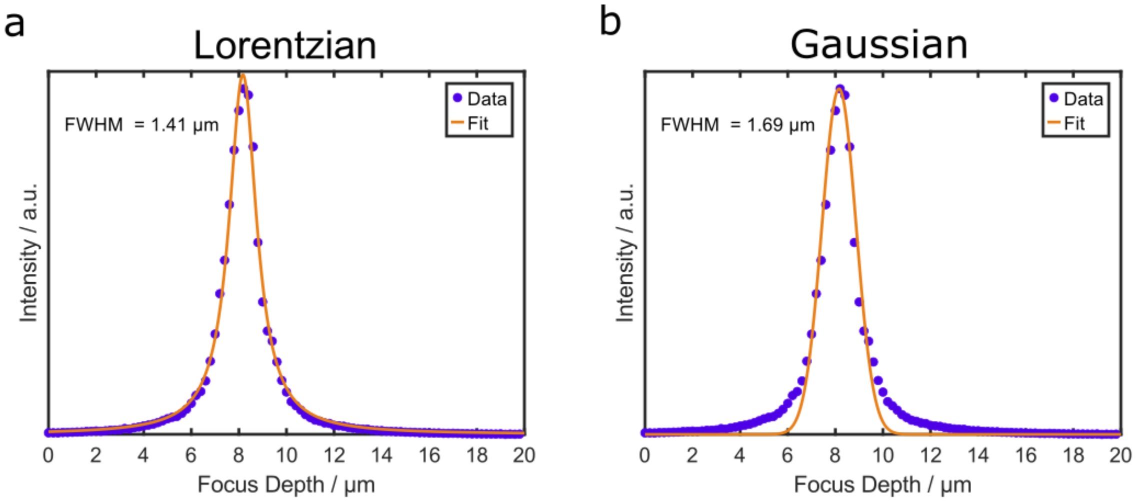

Figure S4: PSF of the 63x/1.0 water immersion objective, evaluated by a confocal through-plane scan through a silicon wafer fitted with a) a Lorentzian (**Equation 8)** and b) a Gaussian. The axial step size was set to 200 nm. A sum filter in the region between 500 cm^{-1} and 580 cm^{-1} was used to integrate the intensity of the silicon signal for each focal position.

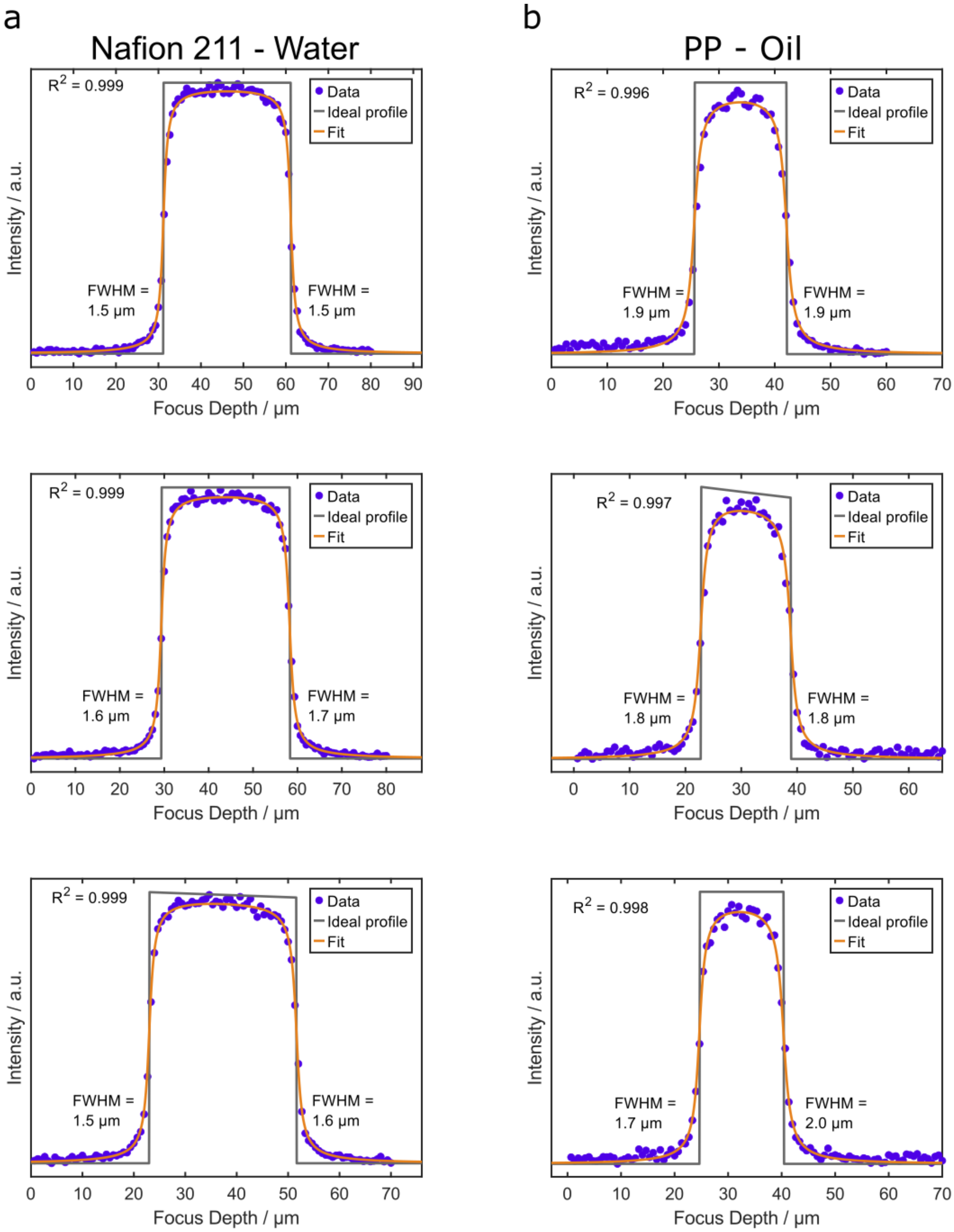

Figure S5: Modelling through-plane scans for negligible index mismatch between sample and immersion medium. a) Three separate depth scans through Nafion 211 with the 63x/1.0 water immersion objective, and b) through-plane scans through PP with the 63x/1.4 oil immersion objective. The step size was set to 666 nm for both cases. Sum filters of 700 - 760 cm^{-1} for Nafion and 790 – 840 cm^{-1} for PP were used for integrating the signal over depth. Fitting the raw data with the model automatically calculates the interface positions and the

attenuation coefficient, and shows the convolution result as a fit superimposed on the raw data points. FWHM indicates the axial resolution at the upper and lower interfaces of the samples. The coefficient of determination was calculated according to **Equation 6.**

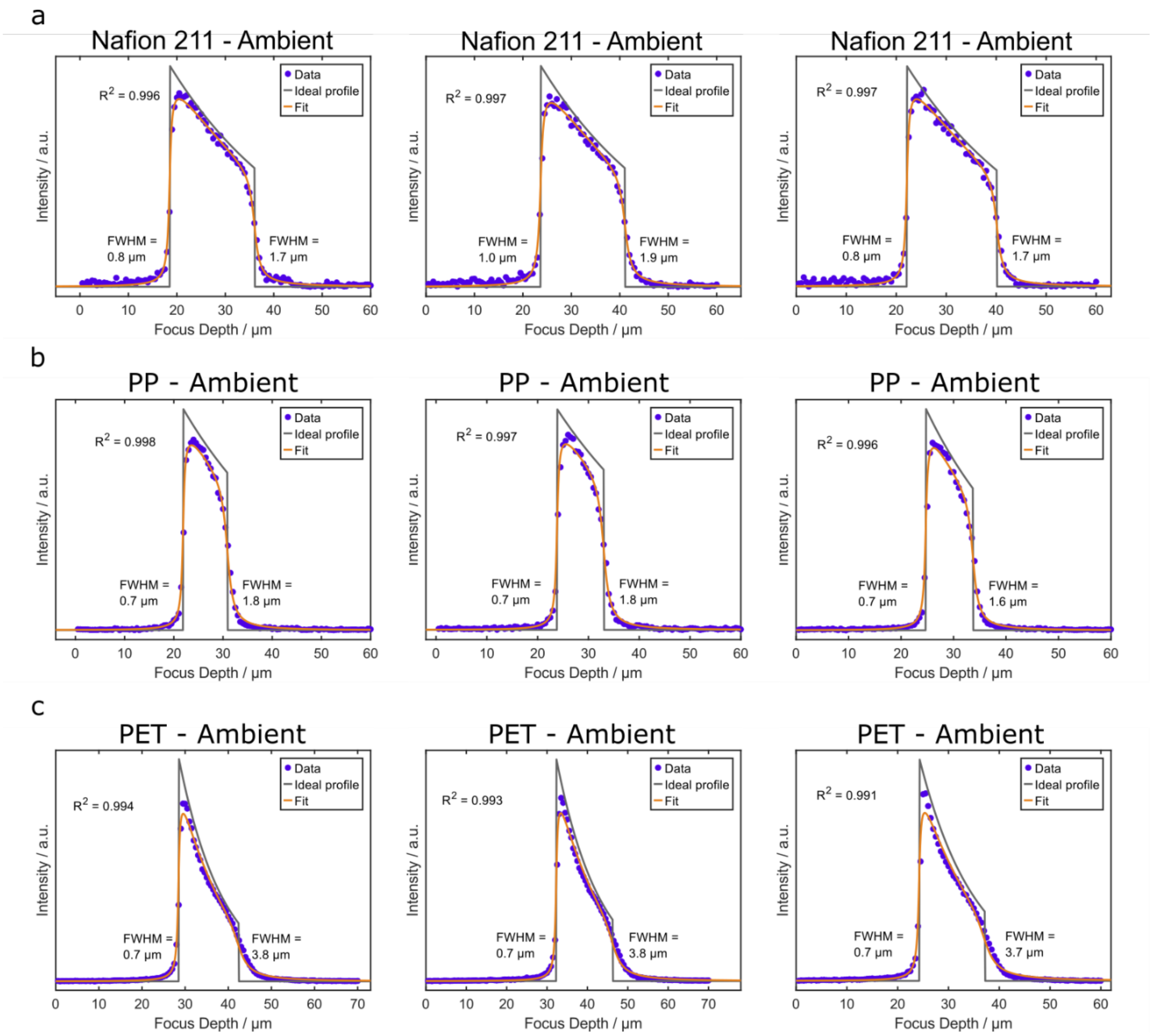

Figure S6: Modelling through-plane scans for significant index mismatch between sample and immersion medium. Three independent depth scans acquired with the 100x/0.9 metallurgical objective through a) Nafion 211, b) PP, and c) PET. The step size was set to 500 nm for all through-plane scans. Sum filters of 700 - 760 cm^{-1} for Nafion, 790 – 840 cm^{-1} for PP, and 1570 – 1650 cm^{-1} for PET were used for integrating the signal over depth. Fitting the raw data with the model automatically calculates the interface positions and the attenuation coefficient and shows the convolution result as a fit superimposed on the raw data points. FWHM indicates the axial resolution at the upper and lower interfaces of the samples. The coefficient of determination was calculated according to **Equation 6.**

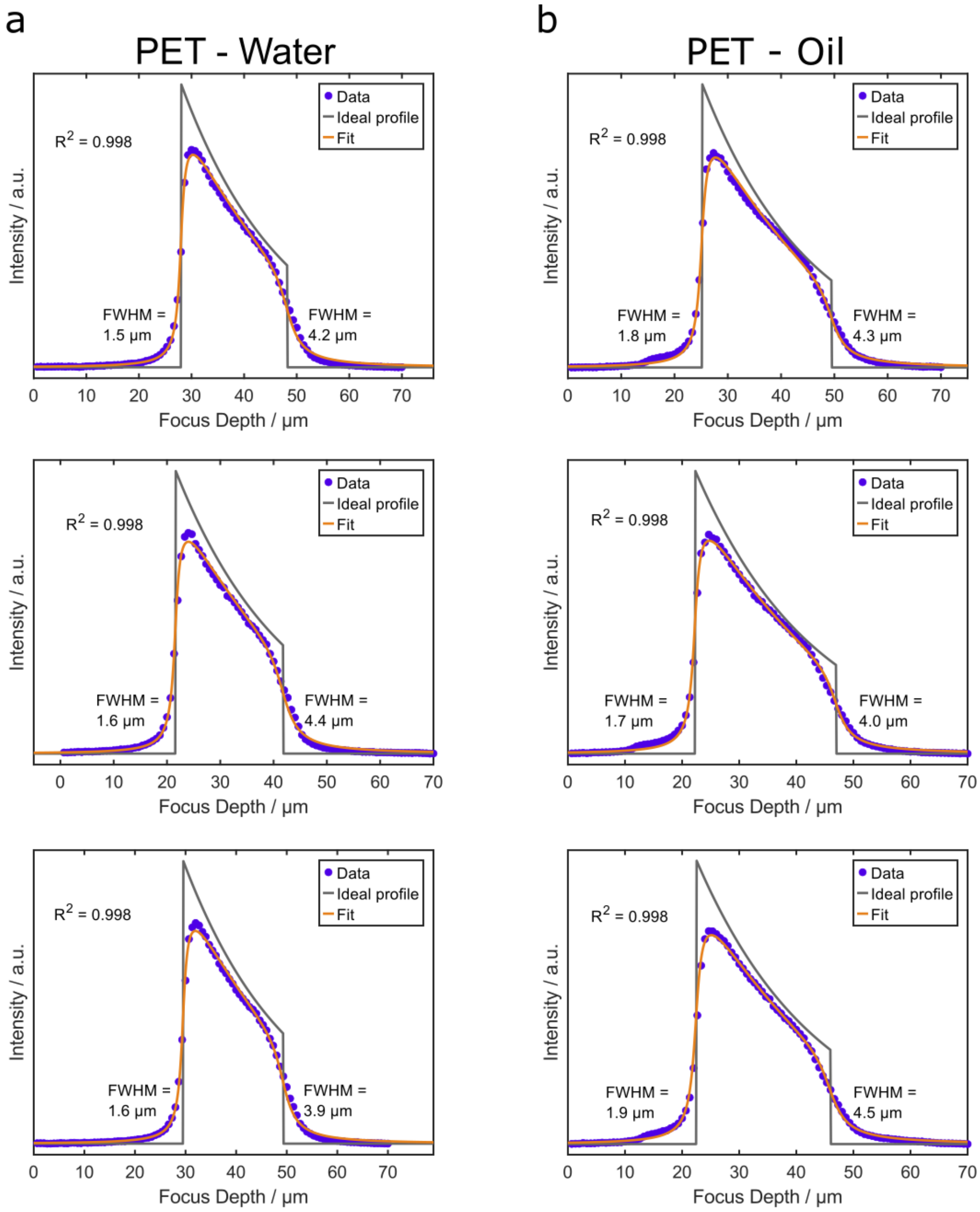

Figure S7: Modelling through-plane scans for significant index mismatch between sample and immersion medium. Three independent depth scans through PET with the a) 63x/1.0 water immersion objective and the b) 63x/1.4 oil immersion objective. The step size was set to 666 nm for both cases. Sum filters of 1570 - 1650 cm^{-1} for water immersion and 1602 – 1672 cm^{-1} for oil immersion were used for integrating the PET signal over depth. Fitting the raw data with the model automatically calculates the interface positions and the attenuation coefficient, and shows the convolution result as a fit superimposed on the raw data points. FWHM indicates the axial resolution at the upper and lower interfaces of the samples. The coefficient of determination was calculated according to **Equation 6.** The second interface of the measurement b) in oil immersion has been set manually.

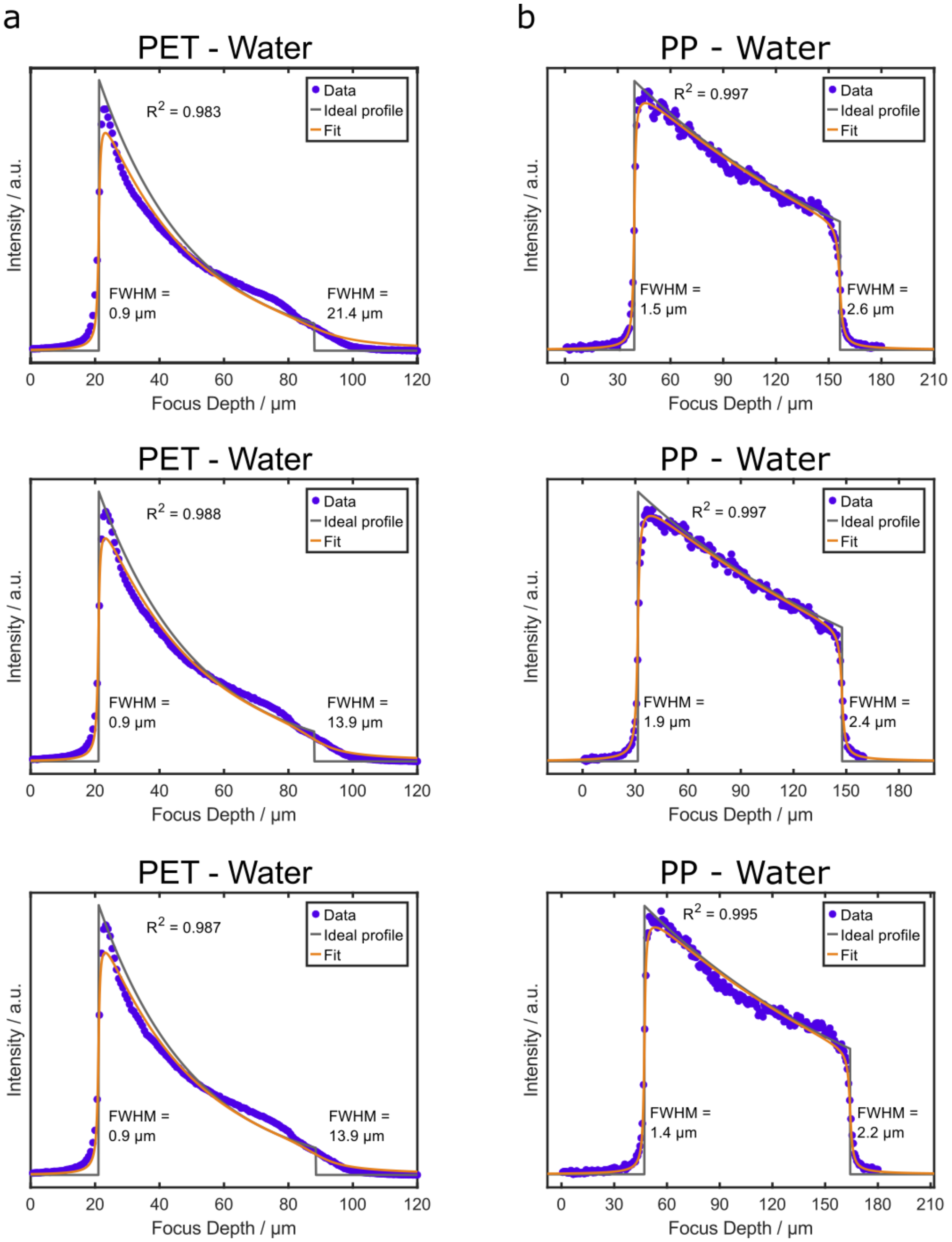

Figure S8: Modelling through-plane scans for polymer films with significant thickness. Depth scan acquired with the 63x/1.0 water immersion objective through a) PET (78µm) and b) PP (135 µm). The step size was set to 666 nm for all through-plane scans. Sum filters of 790 – 840 cm^{-1} for PP, and 1570 – 1650 cm^{-1} for PET were used for integrating the signal over depth. Fitting the raw data with the model automatically calculates the interface positions and the attenuation coefficient, and shows the convolution result as a fit superimposed on the raw data points. FWHM indicates the axial resolution at the upper and lower interfaces of the samples. The coefficient of determination was calculated according to **Equation 6.** The second interface of the measurement of a) PET has been set manually.

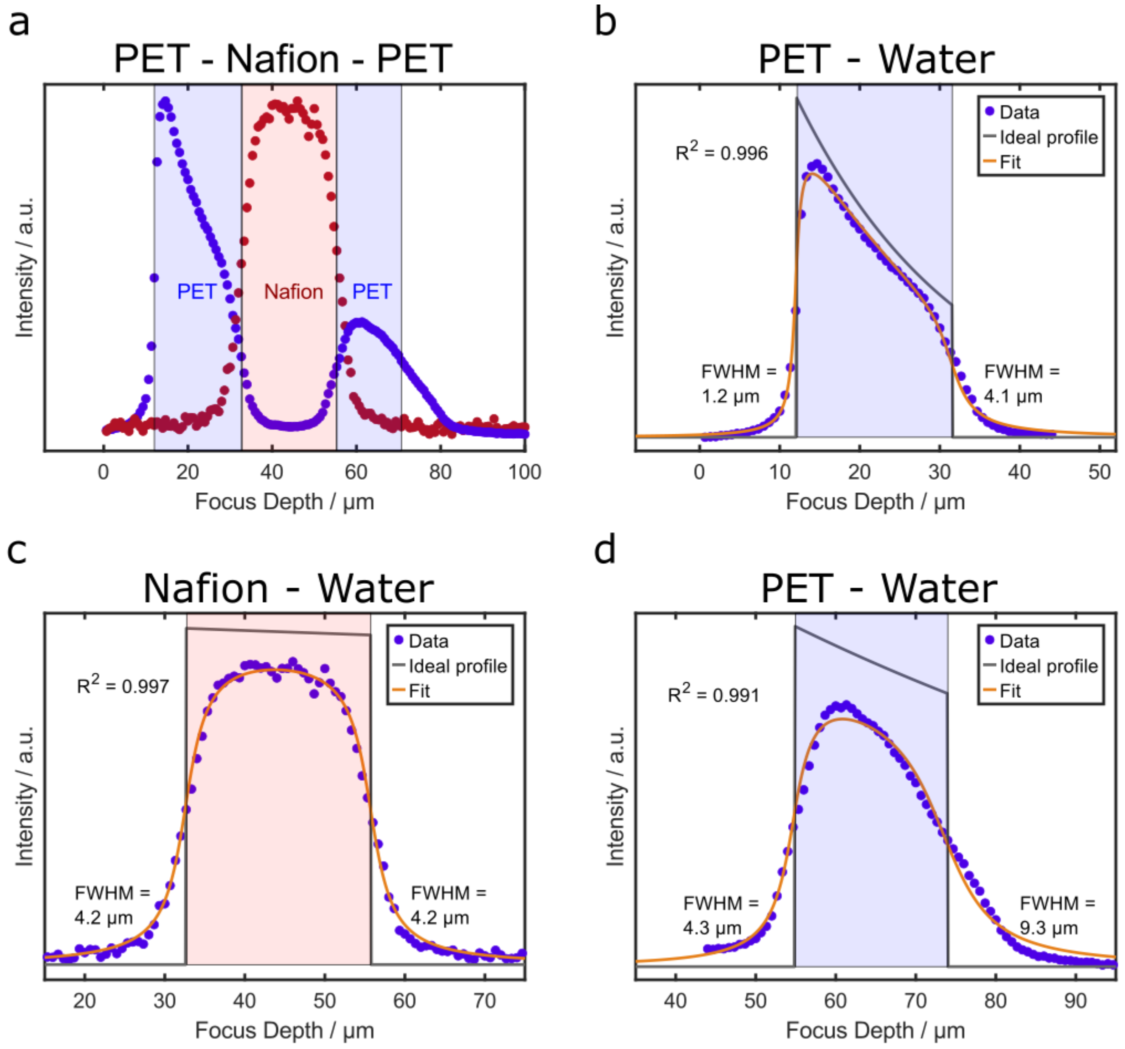

Figure S9: Modelling a through-plane scan for a multi-layered sample consisting of PET with a Nafion 211 interlayer. a) Integrated intensity of the Nafion and PET component signal over focus depth acquired with the 63x/1.0 water immersion objective using a sum filter of 720 - 740 cm^{-1} for Nafion (represented in red) and 1570 – 1650 cm^{-1} for PET (represented in blue). The shaded areas are added as a guide to the eye to indicate the different layers. Modelling the through-plane scan of b) the upper PET layer, c) the Nafion 211 interlayer, and the d) PET bottom layer. The x-axis intervals of b-d) relates to the x-axis of a). Shaded background colors indicate the interfaces between adjacent layers and their respective fits. The step size was set to 666 nm for all through-plane scans. Fitting the raw data with the model automatically calculates the interface positions and the attenuation coefficient, and shows the convolution result as a fit superimposed on the raw data points. FWHM indicates the axial resolution at the upper and lower interfaces of the samples. The coefficient of determination was calculated according to **Equation 6.** The second interface of the d) bottom layer of PET has been set manually.

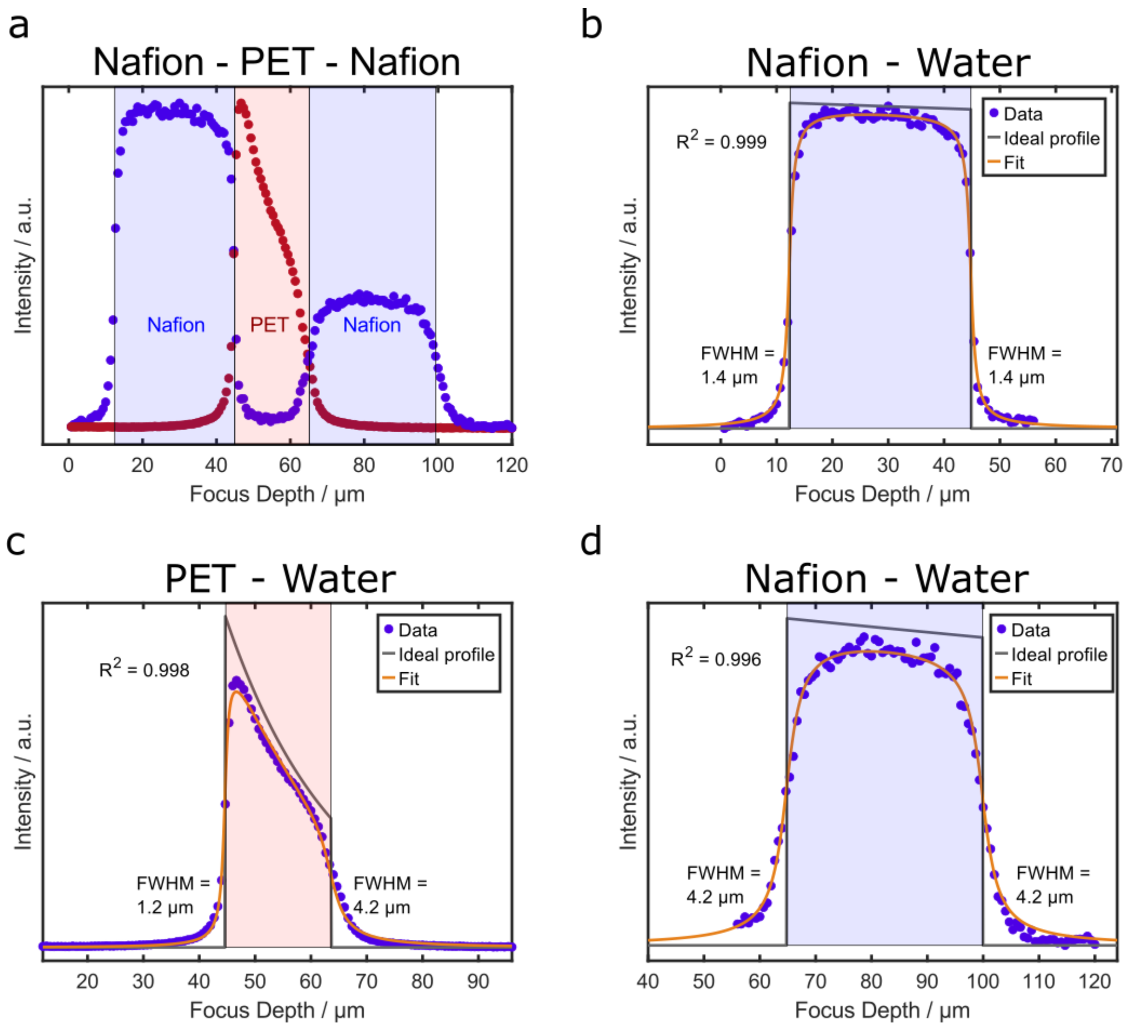

Figure S10: Modelling a through-plane scan for a multi-layered sample consisting of Nafion with a PET interlayer. a) Integrated intensity of the Nafion and PET component signal over focus depth acquired with the 63x/1.0 water immersion objective using a sum filter of 720 - 740 cm^{-1} for Nafion (represented in blue) and 1570 – 1650 cm^{-1} for PET (represented in red). The shaded areas are added as a guide to the eye to indicate the different layers. Modelling the through-plane scan of b) the upper Nafion 211 layer, c) the PET interlayer, and the d) Nafion 211 bottom layer. The x-axis intervals of b-d) relates to the x-axis of a). Shaded background colors indicate the interfaces between adjacent layers and their respective fits. The step size was set to 666 nm for all through-plane scans. Fitting the raw data with the model automatically calculates the interface positions and the attenuation coefficient, and shows the convolution result as a fit superimposed on the raw data points. FWHM indicates the axial resolution at the upper and lower interfaces of the samples. The coefficient of determination was calculated according to **Equation 6.**

Table S1: FWHMs at the first and second interface, the resolution decay per distance, and the attenuation coefficient as independent fit parameters determined by fitting the through-plane scans of the multi-layered Nafion-PET-Nafion sample acquired with the 63x/1.0 water immersion objective and fitting each respective layer with the described model individually (**see 3.2 model development).** Provided are the arithmetic mean and the standard deviation for three independent through-plane scans (**Figure S10).**

**Layer (thickness) and immersion medium**	FWHM [µm] 1st interface	FWHM [µm] 2nd interface	Resolution decay per distance [µm/µm]	Attenuation coefficient µ [$µm^{-1}$]
Nafion (25.4 µm), water	1.37 ± 0.06	1.47 ± 0.12	0.00 ± 0.01	-
PET (23 µm), water	1.33 ± 0.12	4.37 ± 0.21	0.16 ± 0.02	(4.93 ± 0.19) × 10^{-2}
Nafion (25.4 µm), water	4.27 ± 0.12	4.30 ± 0.18	0.00 ± 0.02	-
